# Polymerized Z-**α**-1 antitrypsin leads to lung injury in a murine model of emphysema

**DOI:** 10.1172/jci.insight.199029

**Published:** 2026-08-10

**Authors:** Maria Magallón Serrano, Nazli Khodayari, William Bowers, Edward P. Manning, Xinran Liu, Jungnam Lee, Tammy O. Flagg, Regina Oshins, Aidan Griffin, Sahil Patel, Jorge E. Lascano, Divay Chandra, Susan M. Majka, Irina Petrache, Mark L. Brantly, Karina A. Serban

**Affiliations:** 1 Department of Medicine, Division of Pulmonary, Critical Care, and Sleep Medicine, University of Florida, Gainesville, Florida, USA.; 2 Department of Medicine, Section of Pulmonary, Critical Care, and Sleep Medicine and; 3 Center for Cellular & Molecular Imaging, Electron Microscopy Core Facility, Yale University School of Medicine, New Haven, Connecticut, USA.; 4 Department of Medicine, University of Pittsburgh, Pittsburgh, Pennsylvania, USA.; 5 Department of Medicine, Division of Pulmonary, Critical Care, and Sleep Medicine, National Jewish Health, Denver, Colorado, USA.; 6 Department of Medicine, Division of Pulmonary, Critical Care, and Sleep Medicine, University of Colorado School of Medicine, University of Colorado, Aurora, Colorado, USA.; 7 Department of Molecular Genetics and Microbiology, University of Florida, Gainesville, Florida, USA.

**Keywords:** Cell biology, Genetics, Pulmonology, COPD, Mitochondria, Mouse models

## Abstract

In *α*-1 antitrypsin (AAT) deficiency (AATD), emphysema is classically linked to protease-antiprotease imbalance caused by decreased antiprotease AAT due levels and function. This decrease is secondary to the impaired release of Z-AAT polymers from hepatocytes carrying Pi*Z, E342K mutation in *SERPENA1* gene. Whether the accumulation of Z-AAT polymers in distal lungs contributes directly to emphysema pathogenesis has remained unexplored due to the lack of suitable model systems. We characterized lung injury and airspace enlargement in a Z-AAT–overexpressing murine model. We generated Z-AAT *Serpina1*^Null^ mice overexpressing human (E342K) *SERPENA1* in *Serpina1*^Null^ mice and analyzed pulmonary phenotypes in young and aged animals, complemented by translational studies using primary cells, bronchoalveolar lavage fluid (BALf), and lung tissue from individuals who have never smoked and individuals with AATD. Young Z-AAT *Serpina1*^Null^ mice accumulated Z-AAT polymers in hepatocytes, plasma, and BALf, exhibited spontaneous neutrophilic lung inflammation, increased alveolo-capillary permeability, and premature airspace enlargement, which was worse in older Z-AAT *Serpina1*^Null^ mice. Moreover, Z-AAT polymers accumulated in alveolar type-2 epithelial (AT2) cells and lung macrophages, associated with endoplasmic reticulum (ER) stress, mitochondria dysfunction, and incomplete autophago-lysosomal fusion, which we recapitulated in lung samples from individuals with AATD. These findings support the pathogenic role of Z-AAT polymer accumulation in distal lung epithelium as a driver of epithelial, endothelial, and macrophage dysfunction linked to AATD emphysema.

## Abstract 2

Mouse model lacking normal Alpha-1 Antitrypsin and overexpressing Z Alpha-1 Antitrypsin protein recapitulates human lung injury seen in Alpha-1 Antitrypsin Deficiency.

## Graphical abstract

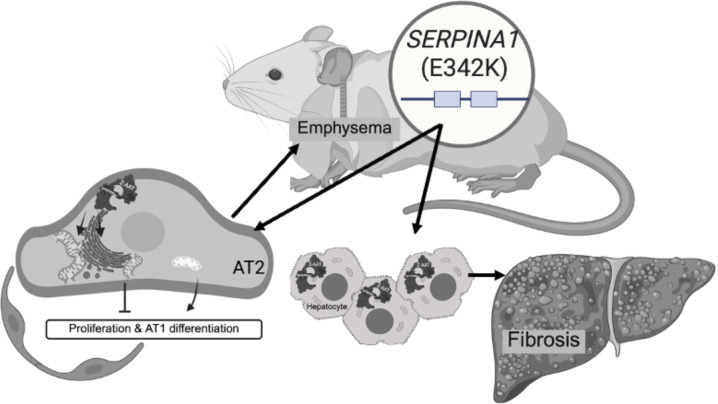

## Introduction

AAT is an abundant circulating protein primarily produced in the liver, with potent inhibitory function against serine proteases, such as neutrophil elastase and proteinase 3, as well as certain metalloproteinases and caspases (1–4). The immune modulatory effects of AAT exerted through antiproteolytic effects are highly dependent on its normal tertiary structure, which, in turn, is vulnerable to any changes in the primary structure of the AAT protein (5, 6). Mutations in *SERPENA1* gene underlying AATD, such as those causing a single amino acid substitution (E342K), result in production of Z-AAT that is prone to polymerization, accumulation, and decreased secretion from the hepatocytes. This accounts for low circulating AAT levels and unbalanced protease burden that predisposes the lung to alveolar destruction, resulting in emphysema, particularly in individuals with tobacco use (7). AAT augmentation via weekly intravenous infusions is the only FDA-approved disease modifying therapy shown to decreases the rate of lung function decline; however, it does not halt the distal lung injury, especially in individuals with AATD with rapid lung function decline (8).

The accumulation of Z-AAT in hepatocytes is responsible for the hepatic pathology in AATD, and increasing evidence suggests that circulating Z-AAT as well as Z-AAT locally produced by lung structural epithelial cells, resident or recruited immune cells, have tissue-specific independent proinflammatory or cellular toxic effects (9–13). Therefore, local Z-AAT accumulation may be, in part, responsible for ongoing lung injury in AATD individuals, even after initiation of augmentation therapy.

We and others have published that the burden of circulating Z-AAT polymers correlate with the severity of AATD-related lung and liver disease (12, 14–16). In hepatocytes the Z-AAT polymers accumulate within the ER lumen and trigger unfolded protein response and ER stress, which, in turn, through IRE1α and ATF6 pathways, cause oxidative stress and mitochondria dysfunction. Similar ER stress, mitochondria dysfunction, and AAT secretion has been shown in other epithelial cells embryologically derived from the gut endoderm, including lung bronchial and AT2 cells derived from pluripotent stem cells (17–23). We have shown that mitophagy, increased interactions between mitochondria and ER — known as mitochondria-associated membranes (MAMs) — and severe imbalances in mitochondrial respiration are mechanistically linked to toxic Z-AAT gain of function in AATD hepatocytes (14). Given the central role of AT2 cells as a facultative progenitor of alveolar type 1 epithelium during repair of the alveolar-capillary unit, a toxic effect of the intrinsic or circulating Z-AAT polymers may contribute to additive emphysema risk in AATD (24, 25) and exacerbate tissue loss following injury, including cigarette smoke (CS) exposure. Moreover, *SERPENA1* expression was increased within primary AT2 cells from individuals with AATD, and it was associated with transcriptional markers of ER and mitochondrial stress (9, 26).

However, a direct role for Z-AAT in the lung injury during the pathogenesis AATD has not been demonstrated. This is, in large part, due to a paucity of transgenic mice available to model both AAT loss of function (low circulating AAT levels) and the accumulation of Z-AAT polymers seen in AATD (27). Indeed, the *Serpina1*^Null^ mice lacking all 5 murine paralogs and the *SerpinA*^Null^ ferrets recapitulated the complete loss of AAT protein, but both lack mutant AAT, and, as such, are devoid of Z-AAT polymers necessary to model AATD (28–30).

Here, we report a murine model of AATD, the Z-AAT *Serpina1*^Null^ strain obtained by microinjecting a transgenic construct containing the full-length DNA of human mutant (E342K) *Serpina1* under its hepatocyte and macrophage endogenous promoters into fertilized *Serpina1*^Null^ oocytes. Z-AAT *Serpina1*^Null^ hemizygous mice, carrying 5–6 copies of the transgene, recapitulated both the AAT loss- and the Z-AAT gain-of-function characteristic of the most common severe form of human AATD. We then characterized the lung and liver phenotype of these transgenic mice, which developed spontaneous age-affected airspace enlargement and other cellular and tissue features that recapitulate AATD pathobiology.

Some of the results of these studies have been previously reported in the form of abstracts (31, 32).

## Results

### Generation and phenotypic characterization of Z-AAT Serpina1^Null^ murine model.

**Figure 1:**
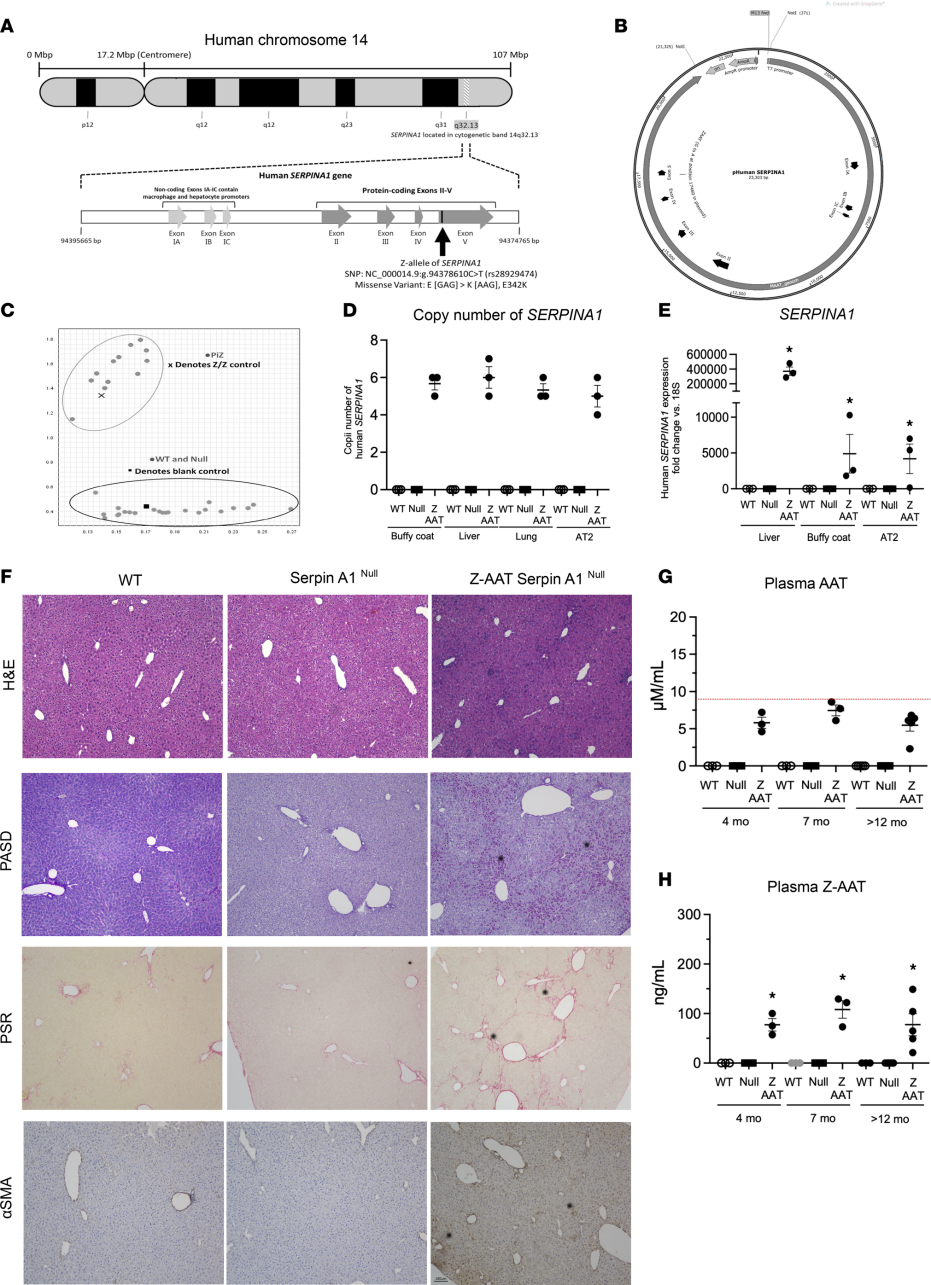
Generation and phenotypic characterization of Z-AAT *Serpina1*^Null^ murine model. Graphic representation of (**A**) the human *SERPENA1* gene on the chromosome 14, q arm, position q2.13. Note the SNP NC-000014.9:g.94378610 C>T (rs 28929474) in the exon V leading to the E [GAG] > K [AAG], E342K mutant AAT. (**B**) the 23-kb pMA-pHuman-Serpina1 construct harboring the glutamic acid to lysine substitution at residue 366 (E366K, also designated 342; G to A; rs 28929474) (**C**) Genotype identification of the Z-AAT *Serpina1*^Null^ versus WT mice using SNP Genotyping TaqMan allelic discrimination assay. (**D**) Somatic integration of pMA-pHuman-Serpina1construct as demonstrated by equal, 5–6 copy number in the liver, lung, buffy coat and AT2 cells of Z-AAT *Serpina1*^Null^ mice (**E**) Human *SERPENA1* gene expression in the liver, lung, buffy coat, and AT2 cells of Z-AAT *Serpina1*^Null^ mice relative to 18S control gene (**F**) Representative H&E, Periodic Acid Shiff globules after treatment with Diastase (PASD), Picro-Sirius Red (PSR) staining and α-smooth muscle actin (α-SMA) images of liver tissue from 4-mo old WT, *Serpina1*^Null^ and Z-AAT *Serpina1*^Null^ mice; scale 100 μm. Note increased PASD, PSR, and α-SMA staining (marked with *) in Z-AAT *Serpina1*^Null^ mice. (**G** and **H**) Human AAT measured by nephelometry (**G**) and human Z-AAT polymers (**H**) measured by custom-made ELISA with specific 2C1 antibody (Hycult) against Z-AAT polymers in the plasma of 4-, 7- and greater than 12-month-old Z-AAT *Serpina1*^Null^ versus *Serpina1*^Null^ and WT mice. The red line, panel **G** represents the upper level (8 μM/mL) of serum AAT in Pi*ZZ individuals. Data are presented as mean ± SD, 1-way ANOVA, followed by Tukey’s multiple comparisons, ^#^*P* < 0.05 versus 4-mo-old same genotype mice, *P < 0.05 versus same age WT mice.

In individuals with AATD, *SERPENA1* (serine protease inhibitor, clade A, member 1 cluster) gene encoding for normal serine protease inhibitor AAT, harbors a single point mutation, E342K (Figure 1A), resulting in mutated Z-AAT protein. Z-AAT is prone to polymerization within the hepatocytes and other epithelial cells embryologically derived from the gut endoderm, with some Z-AAT polymers being secreted into the blood stream (33). Therefore, we designed a 23-kb pMA-pHuman-Serpina1 construct (Figure 1B) that contains the entire human *SERPENA1* gene under its endogenous hepatocyte and macrophage promoters plus 5 kB of the 5’ and 3 kb of the 3’ flanking genomic DNA sequences. The construct also contains the glutamic acid–to–lysine substitution at residue 366 (E366K, also designated 342; G to A; rs 28929474) (34). At the Jackson Laboratory, oocytes from *Serpina1*^Null^ female mice were harvested and microinjected with our construct. Embryos were transferred into pseudo-pregnant females, and the transgenic litters (strain #412938) were backcrossed more than 3 time and maintained on a *Serpina1*^Null^ background (Supplemental Figure 1; supplemental material available online with this article; https://doi.org/10.1172/jci.insight.199029DS1) to ensure germline transmission of the transgene. The offspring are viable and fertile and have a normal lifespan and gender distribution, although decreased litter productivity has been observed in Z-AAT *Serpina1*^Null^ mating pairs. We confirmed their genotype by allelic discrimination assay (Figure 1C) and the somatic integration of our construct by measuring similar copy number of the transgene in various tissues: liver, leukocytes, lung, and AT2 cells (Figure 1D). Z-AAT *Serpina1*^Null^ mice, carrying 5–6 copies of the transgene and having low AAT circulating levels (Figure 1G) were used for experiments.

Although the transgene is under human endogenous promoters, our Z-AAT *Serpina1*^Null^ model recapitulated expected AAT expression pattern, with high *Serpina1* expression in murine hepatocytes, followed by murine circulating monocytes and murine AT2 cells (Figure 1E). Murine H&E-stained liver tissue showed normal hepatocytes, sinusoids, and portal space’s morphology in WT, *Serpina1*^Null^, and Z-AAT *Serpina1*^Null^ mice (Figure 1F), while 4-month-old Z-AAT *Serpina1*^Null^ livers had accumulation of Periodic Acid Shiff globules after treatment with Diastase (PASD; Figure 1F), of collagen fibers on Picro-Sirius Red staining (Figure 1F), and of α-smooth muscle actin (α-SMA) expression (Figure 1F) suggestive of liver fibrogenesis. Moreover, the Z-AAT *Serpina1*^Null^ mice present with low circulating AAT levels, as measured by nephelometry, like Pi*Z AATD individuals (Figure 1G), while expressing a constant high level of circuiting Z-AAT polymers across their lifespan (Figure 1H).

### Z-AAT Serpina1^Null^ mice develop spontaneous lung and systemic inflammation, vascular leak, and protease-antiprotease imbalance.

**Figure 2:**
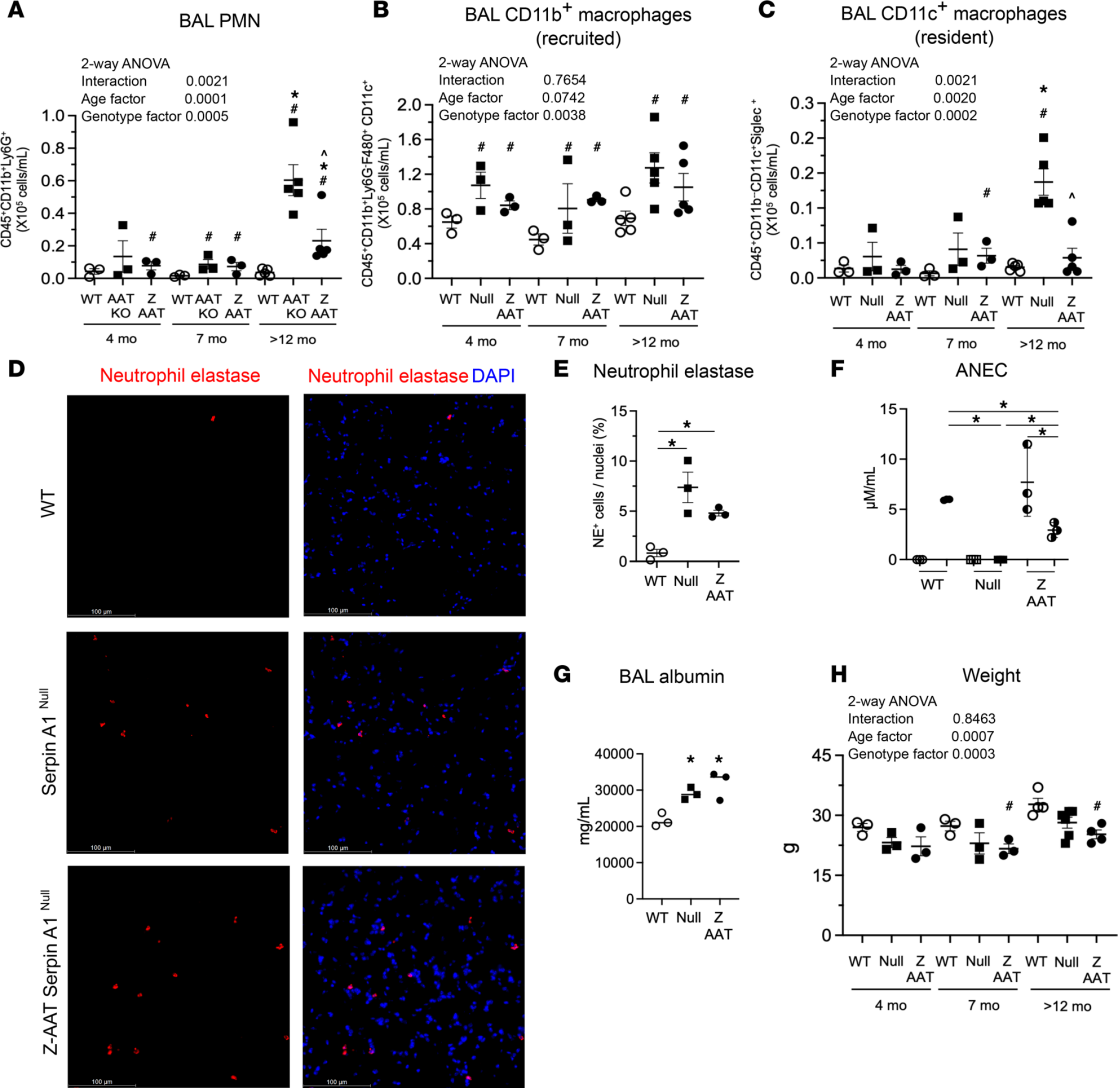
Z-AAT *Serpina1*^Null^ mice develop lung, systemic inflammation, and protease-antiprotease imbalance. (**A**–**C**) Flow cytometry analysis of neutrophils (CD45^+^CD11b^+^Ly6G^+^), recruited monocytes (CD45^+^CD11b^+^Ly6G^–^F4/80^+^),and airway macrophages (CD45^+^CD11b^–^ CD11c^+^SiglecF^+^). (**A**) neutrophils, (**B**) recruited monocytes, and (**C**) airway resident macrophages absolute counts in the BAL fluid of 4-, 7- and greater than 12-month-old Z-AAT *Serpina1*^Null^, *Serpina1*^Null^ and WT mice. (**D** and **E**) Neutrophil elastase (NE) tissue abundance was measured using immunofluorescence and quantified using Image J plug-in. (**D**) Representative immunofluorescence images of 7-month-old WT, *Serpina1*^Null^ and Z-AAT *Serpina1*^Null^ mice; Neutrophil Elastase (NE, red), nuclei (blue, DAPI). scale bar: 100 μm. (**E**) Quantification of NE abundance reported as NE^+^ cells / number of nuclei; 5 fields / mouse, 3 mice per genotype were analyzed. (**F**) Plasma AAT level by nephelometry versus AAT inhibitory activity against NE by absorbance via NE activity assay kit. Note absence of total (open square) and functional AAT (black square) in 7-month-old *Serpina1*^Null^ mice. Lower functional AAT was measured by absorbance via NE activity assay kit (◑) vs. total AAT (◐) measured by nephelometry in 7-month-old Z-AAT *Serpina1*^Null^ mice (**G**) Albumin level (mg/mL) measured by ELISA in BAL fluid of 7-month-old Z-AAT *Serpina1*^Null^, *Serpina1*^Null^, and WT mice. (**H**) Harvest weight (g) of 4-, 7- and greater than 12-month-old Z-AAT *Serpina1*^Null^, *Serpina1*^Null^ and WT mice. Data are presented as mean ± SD, 1-way ANOVA, followed by Tukey’s multiple comparisons or 2-way ANOVA with mixed-effects model (RELM), *P < 0.05 versus 4-mo old same genotype mice, ^#^*P* < 0.05 versus same age WT mice, ^*P* < 0.05 versus same age *Serpina1*^Null^ mice.

We measured baseline airway inflammation in the Z-AAT *Serpina1*^Null^ mice kept in germ-free conditions and quantified total inflammatory cells recruited in the alveolar space using FACS gating strategy, as previously reported (6, 35) (Supplemental Figure 2A). Airway neutrophils (Figure 2A), and CD11b^+^ recruited monocytes (Figure 2B) were increased in 4-months, 7-months, and 12-months in *Serpina1*^Null^ and Z-AAT *Serpina1*^Null^ mice versus WT, control mice. Interestingly, neutrophils, CD11b^+^-recruited monocytes, and airway resident macrophages (Figure 2C) were significantly higher in 12-month-old *Serpina1*^Null^ rather than Z-AAT *Serpina1*^Null^ mice.

We next detected significantly higher neutrophil elastase by immunostaining in the lungs of *Serpina1*^Null^ and Z-AAT *Serpina1*^Null^ versus WT, control mice (Figure 2, D and E). Not only was neutrophil elastase abundance increased, but plasma antineutrophil elastase activity was decreased. Using the Anti Neutrophil Elastase Capacity Assay (ANEC) assay for functional AAT and nephelometry for total AAT, we detected in plasma of WT mice a higher capacity to inhibit human neutrophil elastase than in plasma of Z-AAT *Serpina1*^Null^ mice, while the *Serpina1*^Null^ mice had undetectable antielastase activity and total AAT (Figure 2F). The lung inflammation and imbalanced protease-antiprotease ratio was also associated with significantly higher vascular leak in the BALf of *Serpina1*^Null^ and Z-AAT *Serpina1*^Null^ versus WT, control mice, as measured by albumin extravasation (Figure 2G) and with alveolo-capillary unit morphological changes: thickened basement membrane, collagen accumulation, and fluid extravasation in the interstitial space (Supplemental Figure 3).

Lastly, the weight of 7- and 12-month-old Z-AAT *Serpina1*^Null^ mice was lower than WT and *Serpina1*^Null^ mice, possibly as a result of significant systemic inflammation (Figure 2H).

### Z-AAT Serpina1^Null^ mice develop spontaneous emphysema-like airspace enlargement.

**Figure 3:**
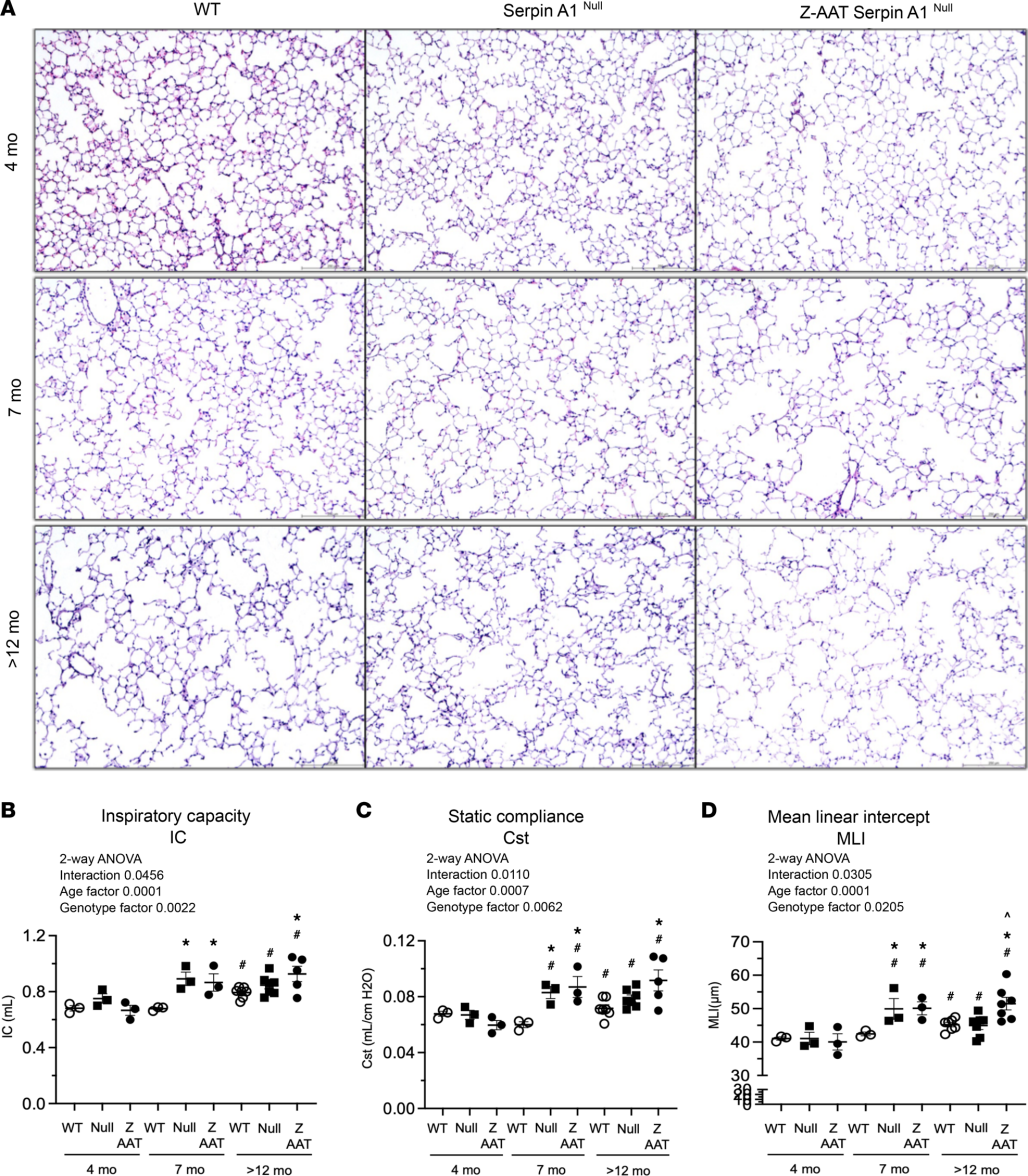
Z-AAT *Serpina1*^Null^ mice develop spontaneous emphysema-like airspace enlargement. (**A**) Representative hematoxylin and eosin (H&E)-stained lung parenchyma sections from 4-, 7- and older than 12-month-old Z-AAT *Serpina1*^Null^, *Serpina1*^Null^ versus WT mice, original magnification, × 10. (**B** and **C**) Murine lung physiology was measured using Flexivent. (**B**) Inspiratory capacity (IC, mL) and (**C**) static compliance (Cst, mL/cm H2O) of 4-, 7- and greater than 12-month-old Z-AAT *Serpina1*^Null^, *Serpina1*^Null^ and WT mice. (**D**) Morphometry measured by Metamorph macro on low melting agarose–inflated and formalin-fixed lungs sampled using stereology methods. Mean linear intercept (MLI, μm, **D**) of 4-, 7- and older than 12-month-old Z-AAT *Serpina1*^Null^, *Serpina1*^Null^ and WT mice. Data are presented as mean ± SD, 2-way ANOVA with mixed-effects model (RELM), ^#^*P* < 0.05 versus 4-mo-old same genotype mice, *P < 0.05 versus same age WT mice, ^*P* < 0.05 versus same age *Serpina1*^Null^ mice.

To determine whether Z-AAT *Serpina1*^Null^ mice develop spontaneous emphysema, we used functional and stereology markers of airspace enlargement. We measured pulmonary mechanics using flexiVent and alveolar architecture using lung morphometry. Brightfield images of representative H&E-stained tissue sections demonstrated normal lung morphology at 4-months old and airspace enlargement in 7- and 12-months old *Serpina1*^Null^ and Z-AAT *Serpina1*^Null^ versus WT, control mice (Figure 3, Ai-vi). We also measured higher inspiratory capacity (IC, Figure 3B) and static compliance (Cst, Figure 3C) in 7- and 12-month-old *Serpina1*^Null^ and Z-AAT *Serpina1*^Null^ versus WT, control mice.

Following flexiVent testing the murine lungs were inflated with low melting agarose solution at constant pressure of 20 cm H_2_O and sectioned using stereology methods for unbiased estimation of alveolar number and alveolar surface. Quantitatively, the mean linear intercept (MLI) was significantly increased in 7- and 12-month-old *Serpina1*^Null^ and Z-AAT *Serpina1*^Null^ versus WT, control mice, with only 12-month-old Z-AAT *Serpina1*^Null^ mice demonstrating progression of emphysema compared with *Serpina1*^Null^ mice at the same age (Figure 3D). The higher static compliance and progressive airspace enlargement in 12-month-old Z-AAT *Serpina1*^Null^ mice suggest that Z-AAT accumulation further worsens lung injury beyond lack of murine AAT, as in *Serpina1*^Null^ mice.

### Z-AAT polymer level in multiple lung compartments of Z-AAT Serpina1^Null^ mice.

**Figure 4:**
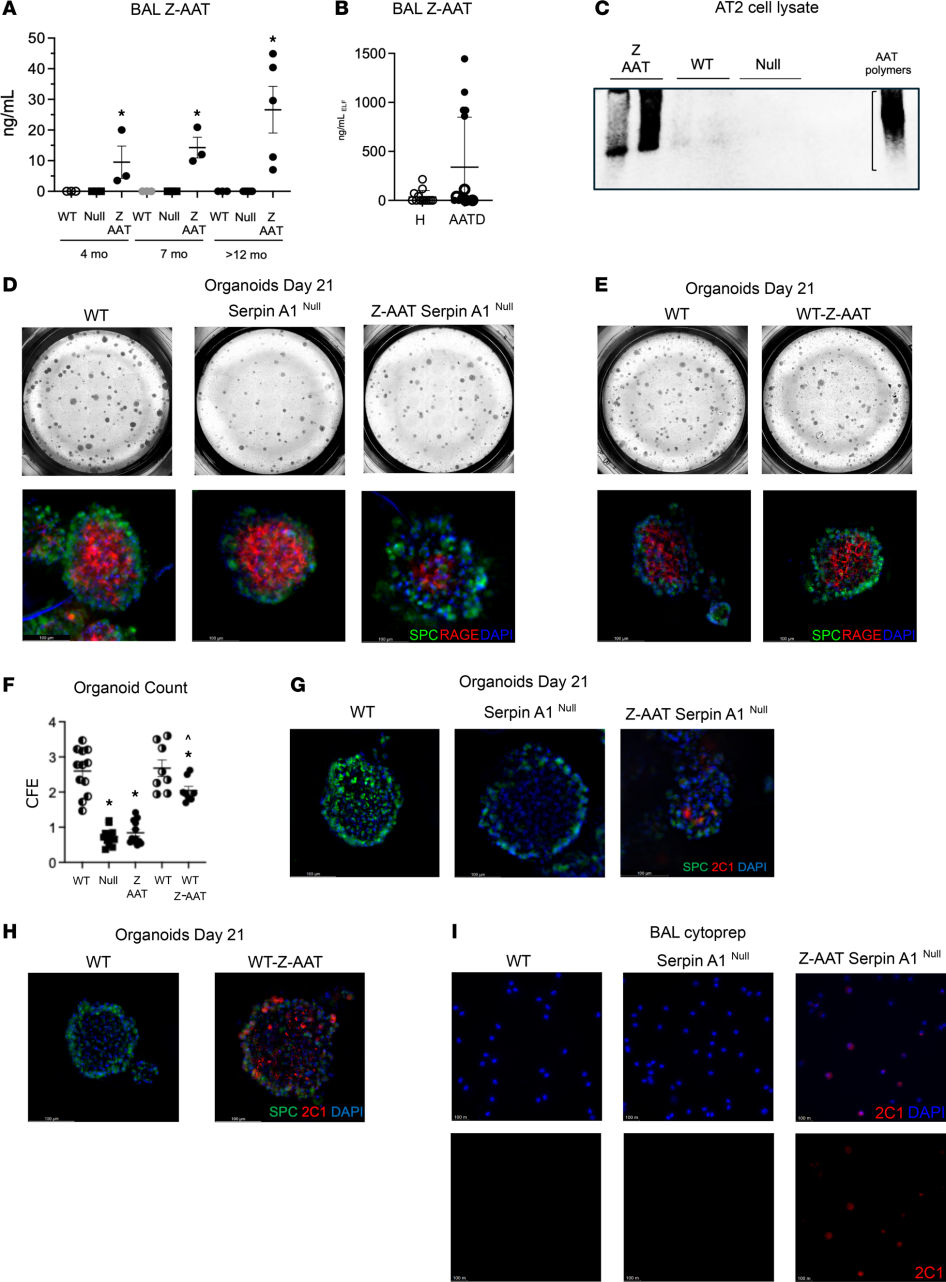
Z-AAT polymers accumulation in lung compartments of Z-AAT *Serpina1*^Null^ mice. (**A** and **B**) Human Z-AAT polymers detected by ELISA using 2C1 antibody against Z-AAT polymers in BAL fluid of 4-, 7- and greater than 12-month-old Z-AAT *Serpina1*^Null^ versus *Serpina1*^Null^ and WT mice (**A**) and of healthy, never smokers (empty circles), Pi*Z AATD (black circles), and exsmoker Pi*Z AATD (larger black circles) individuals (**B**); Z-AAT polymer levels were normalized to epithelial lining fluid (ELF) volume imputed from BALf/plasma urea ratio and BAL volume. (**C**) Representative immunoblot of Z-AAT polymers (75–250 kDa, 2C1 antibody) of primary murine AT2 (EpCam^+^MHCII^+^) cell lysates isolated from Z-AAT *Serpina1*^Null^, *Serpina1*^Null^ and WT mice that were greater than 12-month-old; polymerized AAT (heated M-AAT) served as positive control. (**D**–**F**) Primary murine AT2-derived organoids. Representative whole-well brightfield images (top) of Day 21 organoids derived from primary murine AT2 (EpCam^+^MHCII^+^) cells of greater than 12-month-old Z-AAT *Serpina1*^Null^, *Serpina1*^Null^, WT/Z-AAT, and WT mice cocultured with primary murine fibroblast in Matrigel (**D** and **E**). Organoid quantification (*n* = 4 wells from *n* = 3 mice / genotype) by CFE (**F**). Representative whole-mount immunofluorescent images of D21 organoids stained with anti-SPC (green), anti-RAGE (red, **D** and **E**, bottom), or polymerized Z-AAT (2C1, red, **G** and **H**) derived from AT2 of greater than 12-month-old Z-AAT *Serpina1*^Null^, *Serpina1*^Null^, WT/Z-AAT, and WT mice; scale bar: 100 μm (**D** and **E**, bottom; **G** and **H**) and 20 μm (**D**, top). (**I**) Representative immunofluorescence images of Cytospin slides from the BAL fluid of greater than 12-month-old Z-AAT *Serpina1*^Null^ versus *Serpina1*^Null^ and WT mice stained with 2C1 (red) and DAPI (blue), scale bar:100μm. Data are presented as mean ± SD, 1-way ANOVA, followed by Tukey’s multiple comparisons, or Mann-Whitney test. *P < 0.05 versus age-matched WT mice or healthy (H) individuals, ^ indicates versus age-matched *Serpina1*^Null^ and Z-AAT *Serpina1*^Null^ mice.

We next measured the level of Z-AAT polymers in various lung compartments, using the 2C1 monoclonal antibody that specifically detects naturally occurring human Z AAT polymers. We only measured polymerized Z-AAT in the BALf of Z-AAT Serpina1Null vs. Serpina1Nulland WT control mice, as early as at 4 months of age (Figure 4A and Supplemental Figure 4).

The Z-AAT polymer level in the BALf (26.6 ± 7.6 ng/mL) was lower than plasma level (77.5 ± 22 ng/mL) at every time point, including in greater than 12-month-old Z-AAT *Serpina1*^Null^ mice (Figure 4A and Figure 1H). Our murine model recapitulates the findings seen in 6 of 17 AATD individuals with higher levels of Z-AAT polymers in the BALf (Figure 4B).

Accumulation and secretion of Z-AAT polymers in lung structural cells of individuals with AATD has been elusive and not described in previous murine models of AATD. We detect Z-AAT polymers by Western blotting in whole-cell lysates of unstimulated and nondifferentiated primary murine EpCam^+^MHCII^+^ AT2 cells (Figure 4C) isolated via FACS sorting from greater than 12-month-old Z-AAT *Serpina1*^Null^ mice (Supplemental Figure 5). In a second experiment, FACS-sorted EpCam^+^MHCII^+^ AT2 cells from greater than 12-month-old Z-AAT *Serpina1*^Null^, *Serpina1*^Null^ and WT mice (Supplemental Figure 2B) were cocultured with fetal murine fibroblasts in Matrigel in a 3D organoid model and allowed to proliferate and differentiate for 21 days. Brightfield images of day 21 organoids show lower number of organoids in Z-AAT *Serpina1*^Null^ and *Serpina1*^Null^ versus WT trans wells (Figure 4, D and E – top). During proliferation and differentiation, at day 21, the *Serpina1*^Null^ and Z-AAT *Serpina1*^Null^ organoids demonstrated lower proliferation capacity, as measured by colony forming units (CFE = organoid number / AT2 cells plated * 100, Figure 4F) and expressed less RAGE (Figure 4, D and E – bottom), suggestive of less AT2 proliferation potential and less AT2 to AT1 transition. Moreover, AT2 cells isolated from the Z-AAT mice on WT background (strain #037670) also showed lower CFE and decreased proliferation at day 21 in organoid culture versus AT2-derived organoids from WT littermates (Figure 4, E and F). These findings support an independent and detrimental role of Z-AAT polymers for AT2 proliferation and differentiation, even in presence of normal murine AAT levels as in Z-AAT mice on WT background (strain #037670).

By day 21 the WT-Z-AAT and *Serpina1*^Null^ organoids secreted Z-AAT polymers, as detected by immunofluorescence using 2C1 monoclonal antibody (Figure 4, G and H, and Supplemental Figure 6A).

In a third experiment, after IgG panning AT2 cells isolated from greater than 12-month-old Z-AAT *Serpina1*^Null^, *Serpina1*^Null^, and WT mice were plated on 0.4 μm Corning inserts for 2D monolayer AT1-like differentiation, as previously published (36) (Supplemental Figure 7A). We detected patchy 2C1 staining, suggesting Z-AAT polymers production by day 9 in the *Serpina1*^Null^ AT1-like monolayer (Supplemental Figure 7B).

Moreover, similar to recently published data in lung immune cells of individuals with AATD, we detected, using immunofluorescence, Z-AAT polymers within the airway macrophages of greater than 12-month-old Z-AAT *Serpina1*^Null^ versus *Serpina1*^Null^ and WT mice (Figure 4I and Supplemental Figure 6B).

### Z-AAT polymers accumulation in AT2 cells is associated with mitochondria dysfunction and ER stress in Z-AAT Serpina1^Null^ mice.

Within the hepatocytes of individuals with AATD and murine models of Z-AAT accumulation in the liver, Z-AAT polymerizes in the lumen of the ER, causing ER fragmentation, ER-stress, and activation of the unfolded protein response, resulting in oxidative stress and mitochondria dysfunction (14, 34).

**Figure 5:**
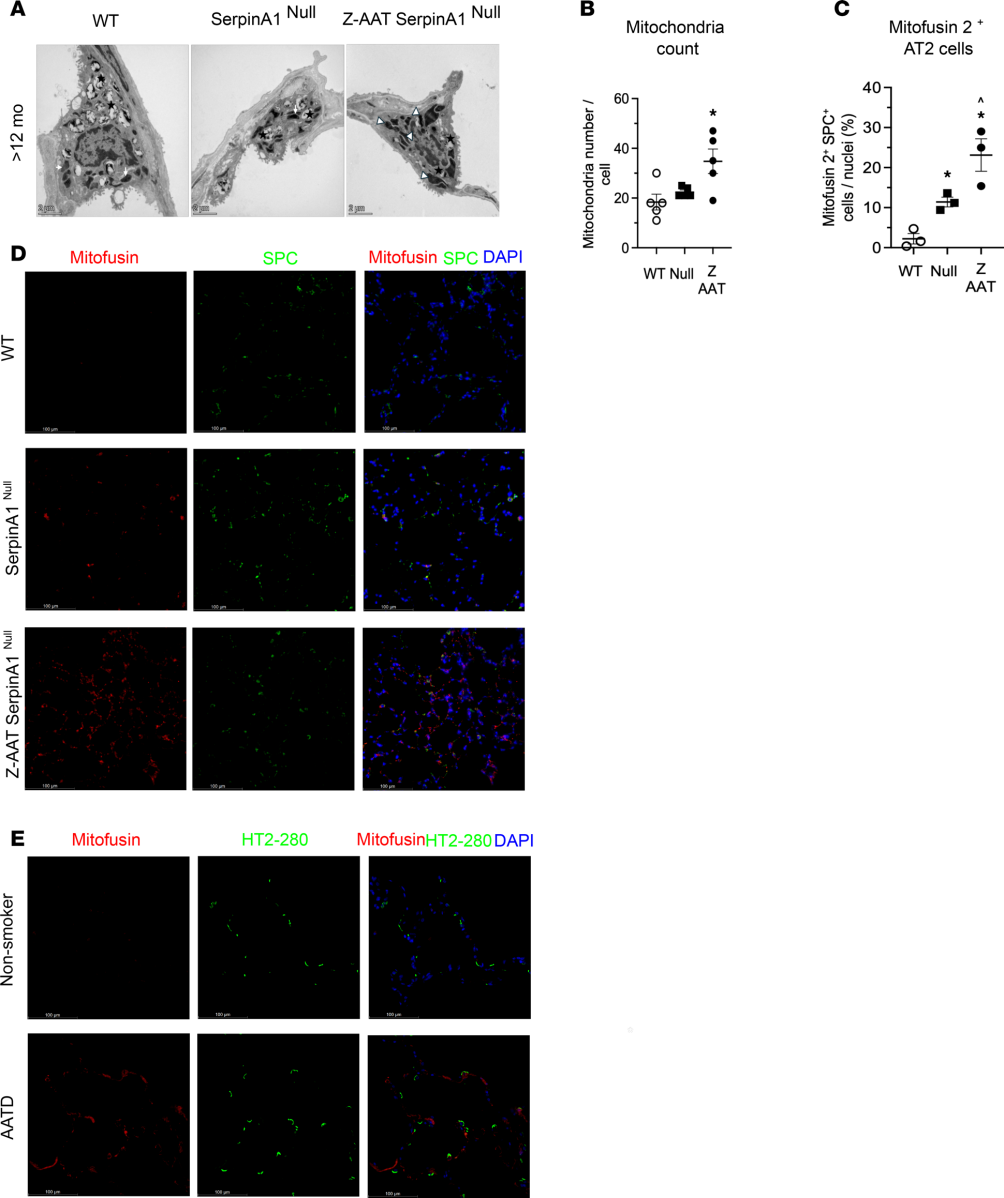
Z-AAT polymers accumulation in alveolar type-2 epithelial cells is associated with mitochondria dysfunction in Z-AAT *Serpina1*^Null^ mice and AATD lung. (**A**) Representative TEM images of in situ murine AT2 of Z-AAT *Serpina1*^Null^, *Serpina1*^Null^ and WT mice; scale bar: 2 μm. Note higher numbers of fused mitochondria (white arrows head), but unchanged number of surfactant-containing lamellar bodies (black star) in greater than 12-month-old Z-AAT *Serpina1*^Null^ versus normal mitochondria (white arrow) *Serpina1*^Null^ and WT mice. (**B**) Number of mitochondria per AT2 cell quantified by manual counting of 5 different TEM images per mouse. (**C** and **D**) Quantification of Mitofusin 2 (red) and pro-SPC (green) and representative immunofluorescence in lungs of 7-month-old Z-AAT *Serpina1*^Null^, *Serpina1*^Null^ and WT mice. Colocalization Mitofusin 2 and pro-SPC (yellow) and quantified by ImageJ analysis of 5 different fields /slide from 3 different mice / genotype. Scale bar: 100 μm. (**E**) Representative immunofluorescence images of healthy, never smoker and AATD lungs stained for Mitofusin 2 (red, mitochondrial marker), HTII-280 (green), and DAPI (nuclei); scale bar: 100 μm. Data are presented as mean ± SD, 1-way ANOVA, followed by Tukey’s multiple comparisons, or Mann-Whitney test *P < 0.05 versus age-matched WT mice.

To study ER morphology, mitochondria abundance, and localization we first evaluated the lungs of greater than 12-month-old Z-AAT *Serpina1*^Null^, *Serpina1*^Null^, and WT mice after fixation with chilled 4% paraformaldehyde / 2.5% glutaraldehyde and analyzed by transmission electron microscopy (TEM). We noticed dilated tubular smooth ER in the AT2 of old Z-AAT *Serpina1*^Null^ versus *Serpina1*^Null^ and WT mice (Supplemental Figure 3). We counted significantly higher number of mitochondria per AT2 surface area in Z-AAT *Serpina1*^Null^, *Serpina1*^Null^ versus WT mice that were greater than 12-months old (Figure 5, A and B). The mitochondria were diffusely localized within the AT2 of Z-AAT *Serpina1*^Null^ mice, while, in the AT2 of *Serpina1*^Null^ and WT mice, they were localized primarily perinuclear. Moreover, mitochondria of Z-AAT *Serpina1*^Null^ mice that were greater than 12-months old showed higher fusion and fission events, as demonstrated by white arrows in Figure 5A. Both Z-AAT *Serpina1*^Null^ and *Serpina1*^Null^ AT2 cells showed fewer lamellar body count (Supplemental Figure 8), but not significantly different than WT AT2 cells.

Mitofusin 2, a mitochondria outer membrane protein that controls fusion, was highly expressed in the AT2 cells (Figure 5C), murine lungs (Figure 5D) of greater than12-month-old Z-AAT *Serpina1*^Null^ versus *Serpina1*^Null^ and WT mice, and in the distal lung of individuals with AATD (Figure 5E).

**Figure 6:**
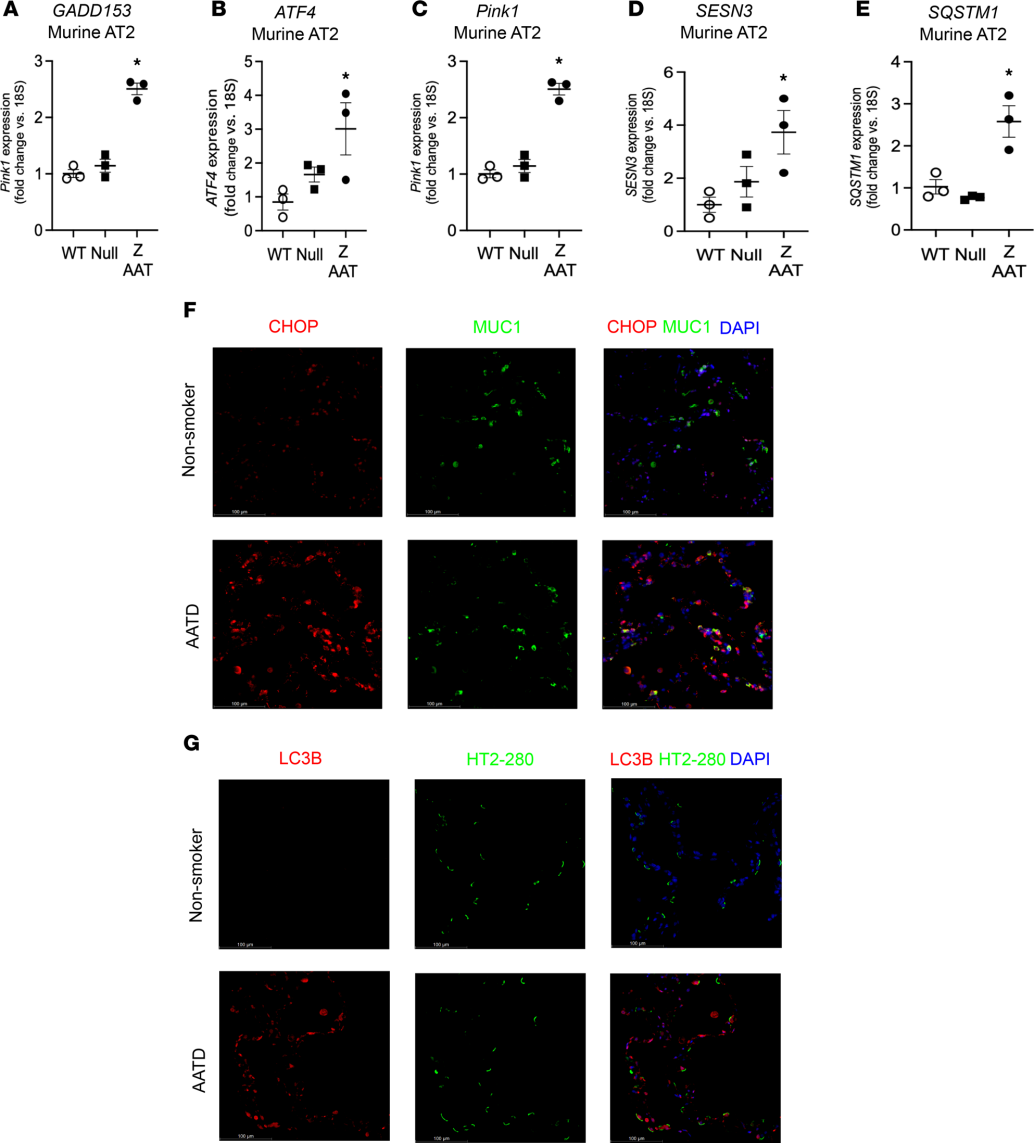
Z-AAT polymers accumulation in alveolar type-2 epithelial cells is associated with ER stress and autophagy in Z-AAT *Serpina1*^Null^ mice and AATD lungs. (**A**–**E**) Expression of indicated genes by RT-qPCR in undifferentiated EpCam^+^MHCII^+^ AT2 cells isolated from greater than 7-month-old Z-AAT *Serpina1*^Null^, *Serpina1*^Null^ and WT mouse lungs. *Gadd153* (ER stress marker, **A**), *Atf4* (unfolded protein response, **B**), *Pink1* (mitophagy, **C**), *Sesn3* (antioxidant and reactive oxygen species defense, **D**), and Sqstm1/p62 (autophagy marker, **E**). (**F** and **G**) Representative immunofluorescence images of healthy, never smoker, and AATD lungs stained for CHOP (red, ER stress marker, **F**), and LC3B (red, autophagy marker, **G**), Muc1 or HTII-280 (green), and DAPI (nuclei); scale bar: 100 μm. Note colocalization (yellow) between CHOP, and LC3B with AT2 cell markers. Data are presented as mean ± SD, *t* test, 1-way ANOVA, followed by Tukey’s multiple comparisons, or Mann-Whitney test *P < 0.05 versus age-matched WT mice.

The unstimulated, nondifferentiated AT2 cells from Z-AAT *Serpina1*^Null^ mice also had significantly higher expression of ER stress, mitophagy, and autophagy markers, as demonstrated by higher expression of *Gadd153* (ER stress, Figure 6A), *Atf4* (unfolded protein response, Figure 6B), *Pink1* (mitophagy, Figure 6C), *Sesn3* (antioxidant and reactive oxygen species defense, Figure 6D), and of *Sqstm1*/p62 (impaired autophago-lysosomal degradation, Figure 6E) versus AT2 cells from *Serpina1*^Null^ and WT mice.

Similarly, we found markers of ER stress and increased autophagy in the distal lung of individuals with AATD, as shown by increased colocalization between CHOP (Figure 6F) and LC3B (Figure 6G) with Muc1 or HT2-280 (AT2 marker), respectively, versus lungs of individuals who have never smoked.

## Discussion

Our Z-AAT *Serpina1*^Null^ murine model developed systemic and pulmonary changes associated with both loss of normal murine M-AAT and presence of mutated human Z-AAT in the systemic circulation and various lung compartments, e.g., BALf, alveolo-capillary membrane, AT2 cells, and airway macrophages. Lack of M-AAT, systemic and BALf Z-AAT, and spontaneous accumulation of Z-AAT polymers in the AT2 cells of Z-AAT *Serpina1^Null^* 12-month-old mice were associated with poor AT2 proliferation and differentiation, ER stress, mitochondria fusion, and mitophagy, alveolo-capillary membrane injury with vascular leak, neutrophil and macrophage recruitment to the alveoli that lead to increase in airspace enlargement in aged Z-AAT *Serpina1^Null^* mice versus *Serpina1^Null^* mice, demonstrating an additive effect of Z-AAT gain of function to the AAT loss of function. This phenomenon has been suspected and described in AATD individuals whose lung disease progresses despite being on augmentation therapy but has been difficult to reproduce in murine models (17, 37, 38). To date, to study in mice the loss of normal M-AAT and protease /antiprotease imbalance that typically trigger emphysema in individuals with AATD, we relied on intratracheal elastase instillation and whole-body cigarette smoke exposure animal models (39, 40). Other animal models of emphysema, e.g., the pallid mouse, the VEGF inhibition model, or the humanized Pi*Z on C57Bl6J background, which express all 5 *Serpina1* murine paralogs plus the human Z-*Serpina1,* all maintained relative levels of native murine AAT expression (27). The first animal models of AAT loss of function were the *Serpina1*^Null^ mouse, lacking all 5 murine paralogs and the *SerpinA*^Null^ ferret model (28, 29). However, because they lack Z-AAT polymer accumulation and a secretion from the hepatocytes and lung cells, these models did not recapitulate the human liver or lung pathology associated with Z-AAT gain of function.

In our model, the Z-AAT polymers were present in circulation as early as 6–8 weeks of age and remain at stable concentration of approximately 80 ng/dL in Z-AAT *Serpina1^Null^* mice across their lifespan. However, in the lung, the contribution of locally secreted Z-AAT polymers from AT2 cells and macrophages is suggested by gradual accumulation of Z-AAT polymers present in the BALf of aged Z-AAT *Serpina1^Null^* mice, while plasma Z-AAT polymers remained stable across all age groups, including the aged Z-AAT *Serpina1^Null^* mice. Moreover, lower Z-AAT polymer levels in BALf than in plasma at every time point suggests in situ, local production and secretion, rather than passive leak through an injured alveolo-capillary membrane, which was present in aged *Serpina1^Null^* and Z-AAT *Serpina1^Null^* mice (13, 41).

Lung inflammation, as measured by the absolute neutrophil, CD45^+^CD11b^+^Ly6G; recruited monocyte, CD45^+^CD11b^+^Ly6G^–^F4/80^+^; and airway macrophage, CD45^+^CD11b^–^ CD11c^+^SiglecF^+^ count was higher in *Serpina1*^Null^ mice than Z-AAT *Serpina1*^Null^ mice at later time points. This suggests that AAT loss of function is directly linked to neutrophil recruitment to the lung and transmigration through the alveolo-capillary membrane, and that the presence of Z-AAT in Z-AAT *Serpina1*^Null^ mice seems to lower but not abrogate proinflammatory cell recruitment. As expected, in both *Serpina1*^Null^ and Z-AAT *Serpina1*^Null^ mice, the plasma antineutrophil elastase activity was decreased, as demonstrated by absent AAT level and activity in the *Serpina1*^Null^ mice and by significant difference between AAT level measured by nephelometry and the ANEC assay in Z-AAT *Serpina1*^Null^ mice. Interestingly, this finding was not associated with lower NE abundance in the lung interstitium in the Z-AAT *Serpina1*^Null^ mice, as demonstrated by immunofluorescence. This suggests that locally released Z-AAT does not efficiently inhibit or neutralize NE release in the lung interstitium (4). These findings confirm that the protease-antiprotease imbalance ineffectively blunted by the presence of Z-AAT is an important mechanism driving chronic lung inflammation (42, 43).

In 7-month-old *Serpina1*^Null^ and Z-AAT *Serpina1*^Null^ mice, the airspace enlargement, as measured by IC, Cst, and MLI, was significantly higher than in the WT mice, but not different between *Serpina1*^Null^ and Z-AAT *Serpina1*^Null^ mice. At greater than 12-month old age, the *Serpina1*^Null^ mice had similar airspace enlargement, measured by IC, Cst, and MLI, consistent with persistence of emphysematous changes. Similar early (at 8 months of age) and persistent (at 12.5 months of age) emphysema development was noted by Borel et al. in *Serpina1*^Null^ mice (28). With aging, the greater than12-month-old Z-AAT *Serpina1*^Null^ mice had higher airspace enlargement, as measured by MLI, suggesting a time-dependent effect of Z-AAT polymer accumulation in the distal lung compartments.

Normal AAT has been shown to have antiinflammatory and antiapoptotic effects on several structural and immune lung cells, e.g., endothelial and alveolar macrophages (44–48). These beneficial effects were linked to its antiprotease function and mediated via inhibition of TNF-α–converting enzyme activity, reducing TNF-α secretion, mannose receptor shedding, and improving macrophage efferocytosis and phagocytosis (6, 44, 45). In-vivo and ex-vivo AAT antiapoptotic role in endothelial cells was independent of AAT antiprotease function, mediated via caspase-3 inhibition (1, 2). Interestingly, polymerized Z-AAT had an inconsequential effect on circulating monocytes and resting bronchial epithelial cells from Pi*Z individuals (17, 37, 38, 49). In the latter, the excessive NF-kB activation was mediated via EGF/MAPK signaling and ameliorated by exogenous normal AAT administration, but they did not accumulate Z-AAT polymers and had no exaggerated ER stress compared with bronchial epithelial cells from Pi*M individuals (17). Here, we focused on primary unstimulated murine AT2 cells, 3D organoids, and 2D monolayer cultures differentiated ex vivo from AT2 cells. We show that, at baseline, AT2 cells express higher markers of ER stress, unfolded protein response, response to oxidative stress, mitophagy, and autophagy in Z-AAT *Serpina1*^Null^ mice. This is consistent with reports from 3D cellular models of human alveolar epithelial cells derived from human-induced pluripotent stem cells of individuals with AATD that showed how *Serpina1* expression is associated with cellular phenotypic changes (22, 50). At baseline and when grown in an organoid model or differentiated in a 2D monolayer culture model the Z-AAT AT2 cells express *Serpina1* transcript, secrete Z-AAT polymers and show signs of poor proliferation. Because both *Serpina1*^Null^ and Z-AAT *Serpina1*^Null^ AT2 cells showed decreased ability to proliferate with lower CFE count, it emphasizes the critical role of AAT loss of function in AT2 biology, without diminishing the contribution of polymerized Z-AAT gain of function to AT2 proliferation ability, which we show is also decreased in WT/Z-AAT mice with normal murine AAT expression.

Recently published research identified *TNFa,* *ATF6*, *ATF4*, unfolded protein response regulons, and apoptosis in primary human AT2 and *CXCL8*, *CXCL2,* and *IL-6* in primary human macrophages from individuals with AATD were significantly upregulated when Pi*Z and Pi*M COPD individuals were profiled by single-cell RNA-seq (9, 26). Similarly, we detected abnormal mitochondria localization, increased fusion, higher mitophagy and autophagy in aged Z-AAT *Serpina1*^Null^ AT2 cells. While we have previously shown abnormal mitochondrial morphology and function associated with hepatic expression and accumulation of human Z-AAT in a Serpina1 overexpression in a AAT-sufficient murine model (14), this is the first time when we describe unfolded protein response, ER stress, mitochondrial changes, and Z-AAT polymer accumulation in the undifferentiated AT2 cells. Our findings suggest that mitochondria of Z-AAT *Serpina1*^Null^ AT2 cells are dynamic organelles responding to extracellular (e.g., circulating Z-AAT polymers) and/or intracellular (e.g., Z-AAT accumulation) stress and injury. Additionally, our findings link fusion regulators such as *MFN2* to epithelial cell stress; mitochondrial fusion being previously linked to homeostatic and injurious stimuli (51–53). Moreover, these morphological and function changes in the mitochondria of Z-AAT *Serpina1*^Null^ AT2 were associated with adjacent structural changes in the endothelium and extracellular matrix, supportive of the complex role of AT2 progenitors in the distal lung. These parallels underscore the model’s utility for preclinical studies focused on cell-type–specific stress responses and regenerative failure.

Our Z-AAT *Serpina1*^Null^ model expresses the human Z-AAT transgene constitutively and is useful to investigate hepatic and pulmonary manifestations that develop spontaneously with aging. This is similar in AATD individuals who carry 1 or 2 of the SERPINA1(E342K) alleles and develop spontaneous liver and lung disease even in the absence of environmental exposures throughout their lifetime (54–56). We have not explored a second hit, e.g. cigarette smoke exposure, lower respiratory tract infections, or a high-fat diet in the *Serpina1*^Null^ nor in the Z-AAT *Serpina1*^Null^ models. It is conceivable that our mice will have accelerated lung and hepatic injury, as the human Z-AAT transgene contains all the inducible promoters that are responsive to proinflammatory stimuli during the acute and chronic phases of inflammation. We plan to perform such experiments in conjunction with a therapeutic intervention, such as *Serpina1* editing to reintroduce normal AAT or modulator therapies, like chemical chaperones or RNAi to decrease Z -AAT polymerization.

Taken together, our data demonstrate that, in a murine model of AAT loss- and gain-of-function, Z-AAT polymer accumulation within various lung compartments is additive to the lung injury caused by lack of normal AAT and protease-antiprotease imbalance. This preclinical murine model allows us to investigate, in vivo, the role of AAT loss of function and Z-AAT gain of function in the AT2 cells, endothelial cells, and lung macrophage biology and in the development and progression of emphysema. Therefore, it represents a true and robust preclinical model to test novel therapeutics, beyond AAT supplementation, like Z-AAT polymer chaperons and correctors that could ameliorate ER stress and mitochondria dysfunction, and halt AATD-lung disease progression.

## Methods

### Sex as a biological variable

Our study examined men and women, and similar findings are reported for both sexes. As per ARRIVE guidelines, an equal number of male and female mice were used, and similar findings are reported for both sexes.

### Reagents

Chemicals and reagents were purchased from ThermoFisher Scientific, unless otherwise stated.

### Human participants

Bronchoalveolar lavage cells and fluid was collected from AATD and healthy individuals at University of Florida. Formalin fixed paraffin embedded slides of lung tissue from AATD and healthy individuals were available from University of Florida tissue biobank and National Jewish Health Human Lung Tissue Research Consortium that uses de-identified lung tissue of deceased subjects not used for organ-transplantation. Patient characteristics are shown in Supplemental Table 1.

### Animals

Mouse lines used included C57BL/6J, C57BL/6JTg(SERPINA1*E366K)1Mlb/J, strain #037670 (WT/Z-AAT), and C57BL/6J-*Serpina1^em3Chmu^*/J knockout, strain #035015 (*Serpina1*^Null^) from Jackson Laboratory and our generated Z-AAT *Serpina1*^Null^ mice (strain #412938).

#### Generation of Z-AAT Serpina1^Null^ mice.

At the Jackson Laboratory oocytes from *Serpina1*^Null^ female mice (C57BL/6J-*Serpina1^em3Chmu^*/J knockout, Jackson Laboratory, Strain#:035015) were harvested and microinjected with human mutant (Pi*Z) variant of AAT full-length DNA construct (Genbank ID: NC_000014.9; sequence 694374747 to 94395692) which contains both the macrophage and hepatocyte enhancer-promoter regions. Embryos were transferred into pseudo-pregnant females, and the resulting pups were genotyped for the presence of human *Serpina1*transgenes. Targeted pups were then bred on the background strain *Serpina1*^Null^ for germline transmission. Transgenic Pi*Z mice heterozygous for the human transgenes and their *Serpina1*^Null^ littermates were transferred to University of Florida where they bred under pathogen-free conditions and fed normal rodent chow.

Genotyping was performed at 1–2 months old on the buffy coat pellet obtained after plasma centrifugation in EDTA-coated tubes. The SNP Genotyping (TaqMan, ThermoFisher) allelic discrimination assay was used to check for the presence of mutant (Pi*Z) variant of AAT. Furthermore, phenotyping of Z-AAT AAT *Serpina1*^Null^ mice was completed by measuring AAT plasma levels for human AAT by nephelometry.

#### Airway physiology measurements.

Mice respiratory function was measured using the flexiVent with a module FX module 2 (SCIREQ, Scientific Respiratory Equipment Inc., Montreal, Canada) as previously described (39). Briefly, ketamine-xylazine anesthetized mice were artificially ventilated through a tracheostomy tube while weight-based pressure and volume maneuvers called perturbations were applied to measure inspiratory and expiratory volume and pressure changes. Data was analyzed as per flexiVent FX user manual, SCIREQ and the following respiratory mechanics parameters were reported inspiratory capacity (IC), total resistance (R), elastance (E), compliance (C), tissue damping (G), tissue elastance (H), and static compliance (Cst).

### Bronchoalveolar lavage procedure

BALf harvest was performed as previously described^6^. Briefly, mice were anesthetized in a chamber containing isoflurane vapors followed by confirmation of euthanasia via thoracotomy and aortic transection. Plasma was collected via cardiac puncture and immediately transferred to tubes prefilled with EDTA. The chest cavity was opened to expose the heart and lungs. The right ventricle was perfused with 10 mL of buffered saline solution. After neck dissection and midline tracheostomy the BAL needle was secured in place and 1mL cold sterile saline containing 2mM EDTA was instilled and recovered three times. Cell suspension from all three BAL aliquots were combined and washed twice with a wash buffer (PBS, 9% FBS, 0.5mM EDTA). After the last wash, cells were spun at 300g for 8 min and processed for flow cytometry.

### Lung processing for morphometry

After perfusion the lungs were inflated under gravitation, at a constant pressure of 20 cm H_2_O, with 1.5% low melting agarose. After excision from the chest using blunt scissors, the lungs were dipped into cold-buffered saline until the agarose solution hardened. The lungs were kept in 10% formalin until post-processing for stereology. Before the lungs were prepared for cutting using a tissue slicer, the lung volume was measured using the water displacement method. Inside the tissue slicer cassette, the lungs were sectioned using the bread-loafing approach into slabs of equal thickness. Every third section (randomization) was selected for submission to the Molecular Pathology Core. H&E-stained sections of stereologically selected lung slices were evaluated by unbiased automated lung morphometry, performed as previously described. MLI were analyzed for frequency distribution using Metamorph (Biovision) macros developed by Dr. Rubin Tuder (University of Colorado, Aurora, CO) and Irina Petrache (National Jewish Health, Denver, CO).

### Lung processing for TEM

For tissue fixation the trachea was cannulated, and the lungs were inflated with chilled 4% paraformaldehyde / 2.5% glutaraldehyde (PFA/GA) in 0.1M sodium-cacodylate buffer with 2mM MgCl2, 1mM CaCl2, and 0.25% NaCl (pH=7.25). Inflated lungs were placed on a cutting board with a puddle of fixative, cut into 1mm^3^ pieces, submerged in 3mL PFA/GA fixative for 1 hour at room temperature, and kept overnight at 4C (57). The tissue sections were submitted to the Electron Microscopy Core for sectioning and imaging.

### Lung processing for immunofluorescence

For tissue fixation the trachea was cannulated, and the lungs were inflated using cold 4% PFA. Inflated lungs were immersed in a conical vial of 4% PFA for fixation and left at 4°C overnight. The day following inflation, the tissue was washed three times with PBS and the fixed lung tissue was trimmed and placed in cassettes. A core facility was used to embed the sample in paraffin. Samples were sectioned at a thickness of 4 μm and adhered to a glass slide. Paraffin sections were incubated at 60°C for fifteen minutes, deparaffinized in xylene (3x for 5 minutes), and rehydrated through an ethanol gradient (100% 2x for 3 minutes each, 95% 2x for 3 minutes each, and 80% for 3 minutes). Antigen retrieval was performed using a citric acid-based solution (Vector, H-3300) at 100°C for 20 minutes. A PBS wash was performed for 15 minutes before sections were outlined using a liquid blocking pen (Vector, H-4000). Sections were treated with a permeabilization solution and blocked for 1 hour using 5% BSA in permeabilization solution at room temperature. Immunofluorescence was performed on the sections using antibodies in Supplemental Table 2. Primary antibody was incubated overnight at 4°C in a humidified box, and secondary antibody was incubated for 1 hour at room temperature. Sections were mounted using Prolong Gold Antifade Mounting Medium with DAPI (Invitrogen) and cover-slipped using a #1.5 coverslip. Mounted sections were allowed to cure overnight while protected from light. The slides were imaged the following day and stored long-term at 4°C.

### Alveolar epithelial cell type 2 (AT2) isolation

Murine AT2 isolation was performed as previously described (58). Briefly, the lungs were instilled with dispase (STEMCELL Technologies), dissect out, and digested for 45 min at room temperature. Mechanically teased lung tissue was filtered through 100 and 40 μm strainers and the single cell suspension was washed with MACS buffer. Positive immunoselection using biotinylated anti- CD45, - CD16/32, - Ter119, - CD90.2, and -CD31 antibodies was performed to deplete immune, red blood cells, fibroblasts, and for endothelial cells. The remaining cell suspension was stained with Live/Dead, EPCAM and MHC II before sorting by flow cytometry.

### Flow cytometry and sorting

For total CD45^+^ counts, 100μl aliquot of the BALf was blocked with CD16/CD32 (clone 93, eBioscience, ThermoFisher), stained with CD45 (BD Horizon), and mixed with 123count eBeads (eBioscience). Cells used for total cell counts were stained and fixed without any centrifugation to avoid variability introduced by pelleting and aspirating. Using the absolute concentration of the counting beads added and the ratio of total CD45^+^ events to total bead events, the concentration of CD45^+^ cells was determined (6, 35). For BAL cell differential, the cells were first blocked with CD16/CD32 (eBioscience) and stained with anti-mouse CD45 (30-F11, BD), Ly6G (1A8, Biolegend), CD11c (N418, eBioscience), F4/80 (BM8, eBioscience), CD11b (M1/70, eBioscience), and Siglec-F (E50–2440, BD) antibodies. Flow data, which included a minimum of 10,000 CD45^+^ events for each sample, was collected using FACSymphony A3 (BD) and analyzed using Flowjo. The gating strategy to identify neutrophil, airway macrophages, and blood-derived macrophages is detailed in Supplemental Figure 1.

Before sorting the epithelial cells isolated by negative selection via Miltenyi column were blocked with CD16/CD32 (clone 93, eBioscience), stained with CD306 (EpCAM, clone G8.8, eBioscience), MHCII (clone M5/114.15.2, eBioscience), and Ghost Dye Red 780 (vWR). Live, EpCAM^+^ MHCII^+^ AT2 cells were sorted using FACSAria Fusion (BD). The gating strategy to identify the AT2 cells is detailed in Supplemental Figure 2.

### AT2 organoids generation and maintenance

EpCAM^+^ MHCII^+^ AT2 cells were resuspended with primary murine fibroblasts (1:5 ratio) and mixed with growth factor reduced Matrigel (Corning). The mixture was seeded into a 24- well plate 0.4 μm trans-well inserts (STEMCELL Technologies) in media containing 10 μM Y-27632 (Cayman Chemical) for the first two days. Cultures were maintained in Small Airway Epithelial Cell Growth media (SAGM, Lonza) for 21 days. Media was changed every other day. Organoid proliferation was measure by brightfield microscopy at day 11 and day 21 of culture using Keyence BZX-710 microscope. Whole-well images were stitched then loaded into FIJI/ImageJ for counting.

#### Organoid isolation, fixation, and immunostaining for whole-mount immunofluorescence.

The organoids were retrieved from the Matrigel using ice-cold Cell Recovery Solution (Corning) and transferred to a 24-well plate. The plate was incubated on a horizontal shaker for 60 min at 4°C. Cut and precoated with PBS-BSA solution (1% wt/vol) pipet tips were used to transfer the dissociated organoids in 15mL PBS-BSA-precoated tubes (Corning). Each conical tube was filled with 10 mL of cold PBS and was spun down at 70g for 3 min at 4°C. After carefully removing the supernatant, the organoids were resuspended in 1 mL of 4% PFA using a precoated tip and incubated for 45 min at 4°C. The conical tubes were then filled with 10 mL of ice-cold PBS-Tween (0.1% vol/vol) and allowed to incubate for overnight at 4°C after gently mixing. The next day the organoids were spun down at 70g for 5 min at 4°C, supernatant was removed, and the organoids were blocked in 200-500 μL of PBS-T/5% donkey serum (0.1% vol/vol Triton X-100 (Sigma). The organoids were then transferred to a 24 well plate and divided into different groups for immunostaining. Primary antibodies and secondary antibodies (Supplemental Table 2) were added in 250μL of PBS-T/5% donkey serum and let to incubate overnight. In between primary and secondary antibodies the organoids underwent serial washes with wash buffer. Before mounting the organoids were then stained with DAPI for 15 min at room temperature.

### Whole-mount organoid clearing and mounting

Stained organoids were washed and allowed to settle to the bottom of their wells. The wash was removed and room temperature fructose-glycerol clearing solution was added to the organoids. After allowing the organoids to clear and settle overnight at 4°C, the organoids were mounted in glass bottom microwell dishes (MatTek Life Sci, P35GC-0-10-C) with a square coverslip (MatTek Life Sci).

### AT2 to AT1-like monolayer differentiation

Murine epithelial cells isolation was performed as previously described (36). Briefly, the lungs were instilled with dispase (STEMCELL Technologies), dissect out, and digested for 45 min at room temperature. Mechanically teased lung tissue was filtered through 100 and 40 μm strainers and the single cell suspension was washed with DMEM media supplemented with 0.01% DNase, 1 mM l-glutamine, and AA. Cells were incubated on petri dishes precoated with anti-mouse CD45 and anti-mouse CD16/32 for 2 h at 37C. Non-adherent epithelial cells were removed, resuspended in complete mouse media, and plated in trans-wells filters (0.4 um pore size, Corning) precoated with 50ug/mL laminin (Sigma Aldrich) 1 for 1h at 37C. Cell culture media was changed after 3 days and every other day thereafter. AT1-like monolayers (Supplemental Figure 3) were maintained at 37C in 5% CO2 incubator until day 9 when after washing with PBS the monolayer was fixed with 4%PFA for 30 min.

### Imaging

Brightfield images were acquired on a Keyence BZX-710 and a Leica DMi8 Fluorescent Imager with Thunder using 4X and 20X objectives. Fluorescent images were acquired on a Leica DMi8 Fluorescent Imager with Thunder using 4X, 20X, and 40X objectives. Images were processed in FIJI/ImageJ (59). For transmission electron microscopy the lung sections were examined with a FEI Tecnai G2 Spirit Twin TEM (FEI Corp., Hillsboro, OR) operated at 120 kV and digital images were acquired with a Gatan UltraScan 2k × 2k camera and processed on the Digital Micrograph software (Gatan Inc., Pleasanton, CA) or a Thermo Scientific Talos L120C G2 TEM or the digital images were acquired with a Thermo Scientific Ceta CMOS 4k × 4k camera and Velox software (ver. 3.11). Mitochondrial ultrastructure was analyzed as described previously (60).

### Western blotting

Cells were harvested in MPER lysis buffer. Whole cell lysates were loaded in equal amounts, as determined by BCA protein analysis (Pierce) and Ponceau staining (Supplemental Figure 4). Proteins were separated by SDS-PAGE and transferred onto a PVDF membrane followed by immunoblotting. The chemiluminescent signal was detected using ECL-plus (Amersham, NJ, USA).

Mouse albumin ELISA assay (Bethyl Laboratories / Fortis Life Sciences, TX, USA) was performed as per manufacturer recommendations.

Human Z-AAT polymer custom ELISA-based assay was performed as previously described (61). Briefly, 2C1 antibody (HycultBiotech, Cat # HM2289)- coated ELISA plates, AAT polymer standards, produced by heating M-AAT isolated from plasma at 55˚C for 18 hours, and goat anti-rabbit IgG (H+L)-HRP Conjugate (BioRad, Cat # 1706515) serving as detection antibodies were used to measure human Z-AAT polymers in plasma samples from mice, healthy and AATD individuals. o-Phenylenediamine substrate (Sigma-Aldrich, Cat # P5412-100TAB) in the presence of catalytic hydrogen peroxide and sulfuric Acid (2.5 M) as a stop solution were added to the plate for colorimetric changes detected with a SpectraMax M3 microplate reader (Molecular Devices, San Jose, CA). Regression for the line of best fit was calculated using the 4-parameter logistic setting and interpolation of unknown samples was determined using the SoftMax Pro 7.1 software (Molecular Devices, San Jose, CA). Points on the standard curve and sample values were only considered valid when CVs for each duplicate standard were within 20% of each other.

### Nephelometry

Total human AAT level was measured in the plasma of WT, *Serpina1*^Null^ and Z-AAT *Serpina1*^Null^ mice using in-house standards and controls and the BNII System (Siemens Healthineers, US) (62).

### ANEC

The assay was performed as previously described (62). Briefly, serial dilutions of plasma mixed with methylamine are incubated for 5 min with 0.1M ANEC buffer at 37C and with human neutrophil elastase (ART Biochem) for 5 min at 37C. Chromogenic substrate containing n-metoxysuccinyl-Ala-Ala-Pro-Val-pNitroanilide in DMSO is added before kinetic reading at 405 nm with correction of 490 nm using SPECTRAmax. Points on the standard curve and sample values were only considered valid when CVs for each duplicate standard were within 20% of each other. Functional AAT measured by ANEC is compared with antigenic AAT, measured by nephelometry.

### Statistics

Statistical analysis was performed with GraphPad Prism v10 (La Jolla, California, USA) using unpaired 2-tailed Student *t* test, 2-way ANOVA with mixed-effects model (RELM), 1-way ANOVA with Tukey’s post hoc multiple comparisons test was used, or nonparametric tests, as appropriate. A *P* value less than 0.05 was considered statistically significant.

### Study approval

The human participant study was approved by the institutional review boards at University of Florida (Gainesville, FL, IRB201501133, IRB201501051 and IRB201501133) and National Jewish Health Human Lung Tissue Research Consortium (Denver, CO, HS-572). All animal studies were conducted under the guidance and supervision of the University of Florida Institutional Animal Care and Use Committee (IACUC202200000127) In accordance with NIH regulatory and biosafety protocols.

Graphics were created in Biorender.com.

### Data availability

Data are available in the Supporting data values file or from corresponding author upon request.

## Author contributions

MMS, WB, TF, RO, AG, and SP performed and analyzed experiments. JEL generated unique reagents or samples. EPM, XL, MMS, NK, JL and KAS planned and interpreted experiments and analyses. DC, SMM, IP, MLB, and KAS wrote the manuscript.

## Conflict of interest

The authors have declared no conflict of interest exists.

## Funding support

This work was supported by NIH funding and is subject to the NIH Public Access Policy. Through acceptance of this federal funding, the NIH has been given a right to make the work publicly available in PubMed Central.

- Alpha-1 Foundation Research Professorship (to KAS).
- National Institute of Health 1R01HL166828 (to KAS).
- Alpha-1 Foundation Research award #1037649 (to JL).
- Alpha-1 Foundation Research Award #1249387 (to JEL).

## Supplementary Material

Supplemental data

Unedited blot and gel images

Supporting data values

## Version 1 08/10/2026

Electronic publication
