# Supplementary material for: Polymerized Z-α-1 antitrypsin leads to lung injury in a murine model of emphysema
Source: JCI Insight. 2026 Aug 10;11(15):e199029. doi: 10.1172/jci.insight.199029 (PMC13463610; doi:10.1172/jci.insight.199029)
Supplement: Supplementary file 1 [file jciinsight-11-199029-s116.pdf]

DATA SUPPLEMENT

Supplementary Table 1 Patient demographics and characteristics

Supplementary Table 2 Key resources

Supplementary figure S1– Murine SerpinA1 gene in *Serpina1* ^{Null} mouse

Supplementary figure S2 – Gating strategy to identify inflammatory cells in the WT mouse BAL fluid and primary murine AT2 cells

Supplementary figure S3 – TEM images of the alveolo-capillary unit

Supplementary figure S4 – BAL fluid to plasma Z-AAT polymer ratio

Supplementary figure S5 - AT2 cells immunoblot Ponceau S staining

Supplementary figure S6 – Z-AAT polymers in AT2 organoids and murine macrophages

Supplementary figure S7 – Primary murine AT2 differentiation into AT1-like cells using a trans-well culture system secrete Z-AAT polymers

Supplementary figure S8 – TEM quantification of AT2 lamellar body abundance

Supplementary Table 1

Patient characteristics	Never-smoker	Smoker	AATD
**Tissue explant subjects**	N=3	N=3	N=4
Age, age (Mean ± SD)	52.6 ± 11.85	62.6 ± 0.57	56 ± 6.63
Sex, M (%)	100	100	100
Smoking history, PY (Mean ± SD)	1 ± 1.4	55 ± 35	42 ± 27
**Bronchoscopy subjects**	N=14	N/A	N=17
Age, age (Mean ± SD)	31.1 ± 11.4	N/A	57.2 ± 7.5
Sex, M (%)	71.4	N/A	23.5
Smoking status		N/A	
Never smoker	0		9
Ex-smoker			6
Smoking history, PY (Mean ± SD)	0	N/A	11 ± 7.1
FEV1 (% pred)	106.7± 14.2	N/A	80 ± 17.6

Legend: AATD: Alpha-1 Antitrypsin Deficiency; PY: pack-year;

FEV1: forced expiratory volume in 1 second

Supplementary Table 2

Reagent or Resource	Concentration	Source	Identifier
**Antibodies - Flow Cytometry**			
CD16/CD32 Monoclonal Antibody (93), eBioscience	1:400-1:100	Thermofisher	14-0161-82
Pacific Blue™ anti-mouse Ly-6G Antibody	1:400	BioLegend	127612
CD11c Monoclonal Antibody (N418), APC-eFluor™ 780, eBioscience	1:400	Thermofisher	47-0114-82
CD11b Monoclonal Antibody (M1/70), PerCP-Cyanine5.5, eBioscience	1:400	Thermofisher	45-0112-82
F4/80 Monoclonal Antibody (BM8), PE-Cyanine7, eBioscience	1:400	Thermofisher	25-4801-82
BD Horizon™ BUV395 Rat Anti-Mouse CD45	1:400	BD Biosciences	564279
BD Horizon™ PE-CF594 Rat Anti-Mouse Siglec-F	1:400	BD Biosciences	562757
CD326 (EpCAM) Monoclonal Antibody (G8.8), eFluor™ 450, eBioscience™	1:25-1:100	Thermofisher	48-5791-82
MHC Class II (I-A/I-E) Monoclonal Antibody (M5/114.15.2), Alexa Fluor™ 700, eBioscience™	1:25-1:100	Thermofisher	56-5321-82
Biotin anti-mouse CD45 Antibody	1:100	BioLegend	103104
Biotin anti-mouse TER-119/Erythroid Cells Antibody	1:200	BioLegend	116204
Biotin anti-mouse CD31 Antibody	1:200	BioLegend	102503
Biotin anti-mouse CD90.2 (Thy1.2) Antibody	1:200	BioLegend	105304
Biotin Rat Anti-Mouse CD16/CD32	1:100	BD Pharmingen™	553143
**Antibodies - Western**			
2C1, Alpha-1-antitrypsin, Human, mAb	1:25	Hycult	HM2289
Vinculin (E1E9V) XP® Rabbit mAb (HRP Conjugate)		Cell Signaling Technology	18799S
**Antibodies - Immunofluorescence**			
2C1, Alpha-1-antitrypsin, Human, mAb	1:25	Hycult	HM2289
Anti-Prosurfactant Protein C (proSP-C) Antibody	1:200	Sigma	AB3786
Mouse/Rat RAGE Antibody	1:100	Biotechne R&D Systems	MAB1179
Anti-Podoplanin Antibody, clone 8.1.1		Sigma	MABT1512
Neutrophil Elastase Polyclonal Antibody	1:100	Thermofisher	PA5-115648
CHOP (L63F7) Mouse mAb	1:50	Cell Signaling Technology	2895S
Mitofusin-2 (D2D10) Rabbit mAb	1:100	Cell Signaling Technology	9482S
Anti HT2-280	1:100	Terrace Biotech	TB-27AHT2-280
Goat anti-Rabbit IgG (H+L) Highly Cross-Adsorbed Secondary Antibody, Alexa Fluor™ 488	1:500	Thermofisher	A-11034
Goat anti-Mouse IgG (H+L) Highly Cross-Adsorbed Secondary Antibody, Alexa Fluor™ 488	1:500	Thermofisher	A-11029
Goat anti-Mouse IgG1 Cross-Adsorbed Secondary Antibody, Alexa Fluor™ 488	1:500	Thermofisher	A-21121
Goat anti-Mouse IgM (Heavy chain) Cross-Adsorbed Secondary Antibody, Alexa Fluor™ 488	1:500	Thermofisher	A-21042
Goat anti-Mouse IgG1 Cross-Adsorbed Secondary Antibody, Alexa Fluor™ 568	1:500	Thermofisher	A-21124
Goat anti-Rabbit IgG (H+L) Cross-Adsorbed Secondary Antibody, Alexa Fluor™ 647	1:500	Thermofisher	A-21244
Goat anti-Mouse IgG2a Cross-Adsorbed Secondary Antibody, Alexa Fluor™ 647	1:500	Thermofisher	A-21241
**TaqMan Probes**			
Mouse Serpina1a FAM-MGB		Thermofisher	Mm02748447_g1
Mouse Ddit3 FAM-MGB		Thermofisher	Mm01135937_g1
Mouse Sqstm1 FAM-MGB		Thermofisher	Mm00448091_m1
Mouse Pink1 FAM-MGB		Thermofisher	Mm00550827_m1
Human SERPINA1 FAM-MGB		Thermofisher	Hs00165475_m1
Human DDIT3 FAM-MGB		Thermofisher	Hs00358796_g1
Human SQSTM1 FAM-MGB		Thermofisher	Hs01061917_g1
Human PINK1 FAM-MGB		Thermofisher	Hs00260868_m1
Eukaryotic 18S rRNA Endogenous Control		Thermofisher	4310893E
**Reagents and Chemicals**			
Liberase™ TM Research Grade		Sigma	5401119001
Dispase (5 U/mL)		STEMCELL Technologies	07913
123count eBeads™ Counting Beads		Thermofisher	01-1234-42
Applied Biosystems™ TaqMan™ Fast Advanced Master Mix for qPCR		Thermofisher	4444557
Antigen Unmasking Solution, Citrate-Based		Vector Laboratories	H-3300-250
10x Tris/Glycine Buffer for Western Blots and Native Gels		BioRad	1610771
10x Tris/Glycine/SDS		BioRad	1610772
Ghost Dye™ Red 780		Cytek Biosciences	SKU 13-0865-T100
Anti-Biotin MicroBeads		Milteny Biotec	130-090-485
**Software**			
FlowJo		BD Biosciences	Version 10.10
Metamorph		BioVision	Version
Image Lab Software		BioRad	Version 6.1
ImageJ		NIH	Version 1.54m
Keyence BZ-X Analyzer		Keyence	Version 1.3.1.1
Leica LASX Office		Leica	Version 1.4.7 28921

Supplementary figure S1. Murine SerpinA1 gene in *Serpina1* ^{Null} mouse. All five a-e *SerpinA1* paralogs were deleted via CRISPR-Cas9 in *Serpina1* ^{Null} mouse (C57BL/6J-Serpina1em3Chmu/J knockout, strain #035015) from Jackson Laboratory.

Supplementary figure S2. Gating strategy to identify inflammatory cells in the WT mouse BAL fluid and primary murine AT2 cells. **A**. Single cells of the BAL cell pellet, after the exclusion of debris and doublets were identified by CD45 staining. A sequential gating strategy was used to identify populations expressing specific surface markers: CD11b$^-$ F4/80$^+$ CD11c$^+$ airway macrophages, CD11b$^+$ Ly6G$^+$ neutrophils, CD11b$^+$ F4/80$^+$ CD11c$^+$ monocyte – derived recruited macrophages. **B**. Single cell suspension obtained by enzymatic digestion of mouse lungs was subjected to flow cytometry and sorting after the exclusion of debris and doublets. A sequential gating strategy was used to sort for live, EpCAM$^+$MHCII$^+$ (AT2) cells   FSC - forward scatter, SSC – side scatter.

Supplementary figure S3 – TEM images of the alveolo-capillary unit. Representative TEM images of alveolo-capillary unit in Z-AAT *Serpina1* ^{Null}, *Serpina1* ^{Null} and WT mice; size bar 2 μm. Note thicker basement membrane, accumulation of fibrillar collagen, and fluid leak in the interstitial space in *Serpina1* ^{Null} and Z-AAT *Serpina1* ^{Null} vs. WT mice, accompanied by fussed mitochondria and dilated tubular ER in Z-AAT *Serpina1* ^{Null} mice.

Supplementary figure S4. **BAL fluid to plasma Z-AAT polymer ratio**. Z-AAT levels measured by ELISA using 2C1 antibody in the BAL fluid and plasma of 4-, 7- and older than 12-month-old Z-AAT Serpina1 Null, Serpina1 Null and WT mice. Note the trend in higher BAL/plasma Z-AAT ratio in the older than 12-month-old Z-AAT mice, but not statistically different than 4-, 7-month-old Z-AAT mice. Data are presented as mean ± SD, 1-way ANOVA, followed by Tukey's multiple comparisons.

Supplementary figure S5. AT2 cells immunoblot Ponceau S staining. Immunoblot of whole cell lysates from primary murine AT2 cells of Z-AAT *Serpina1* ^{Null}, *Serpina1* ^{Null} and WT mice stained with Ponceau S to verify equal loading.

Supplementary figure S6. **Z-AAT polymers in AT2 organoids and murine macrophages**. **A**. Representative whole-mount immunofluorescent images of day 21 organoids stained with anti-SPC (green) and 2C1 (red) showing Z-AAT polymers secretion by differentiated AT2 from 3 different Z-AAT *Serpina1* ^{Null} mice, age 7-12-month-old vs. *Serpina1* ^{Null} mice; size bar 100μm **B**. Representative immunofluorescence images of Cytospin slides obtained from the BAL fluid of >12-month-old Z-AAT *Serpina1* ^{Null} vs. *Serpina1* ^{Null} mice after staining with 2C1 antibody (red, Hycult) and DAPI (blue); size bar 100μm.

Supplementary figure S7. **Primary murine AT2 differentiation using a trans-well culture system secrete Z-AAT polymers. A.** Representative immunofluorescence images of Day 9 monolayers stained with an AT1 marker, podoplanin (green) and DAPI (blue) showing tight monolayer formation by differentiated AT2 cells of old WT mice grown in laminin-coated trans-wells. Note incomplete monolayer differentiated from AT2 cells of old Z-AAT *Serpina1* ^{Null} and *Serpina1* ^{Null} mice; scale 100 μm. **B**. Representative immunofluorescence images of Day 9 monolayers stained with 2C1 (red) and DAPI (blue) showing polymerized Z-AAT (red), size bar 100μm.

Supplementary figure S8 – TEM quantification of AT2 lamellar body abundance. Number of lamellar bodies per AT2 cell quantified by manual counting of 5 different TEM images from >12-month-old Z-AAT *Serpina1* ^{Null} vs. *Serpina1* ^{Null} and WT mice. Data are presented as mean ± SD, t-test.

Supplementary Figure S1

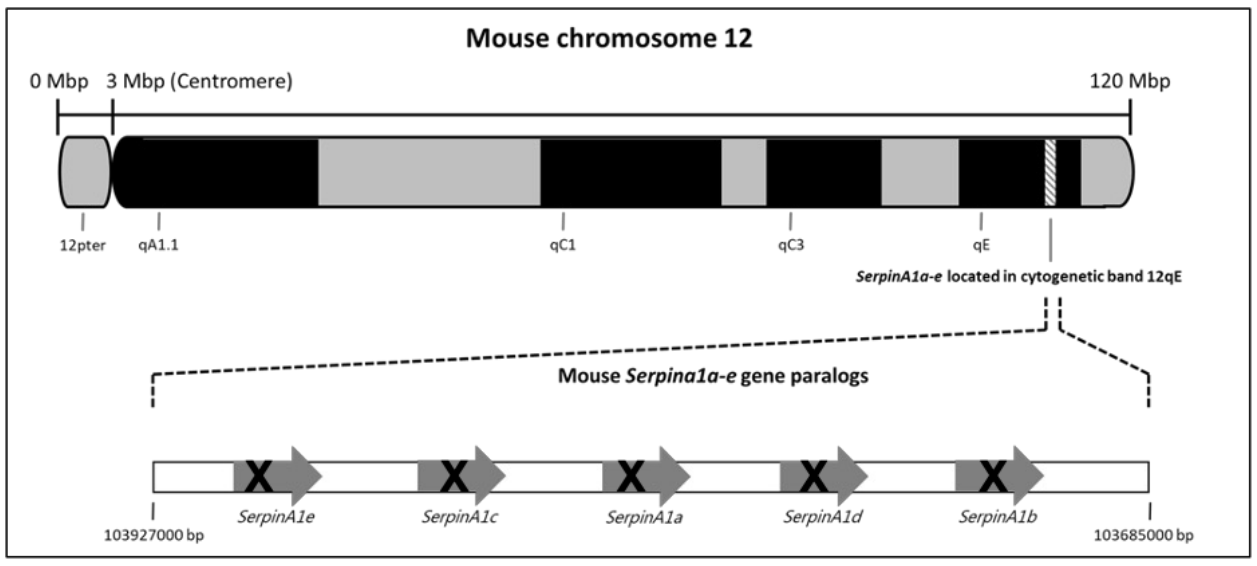

Supplementary figure S1. Murine SerpinA1 gene in *Serpina1* ^{Null} mouse. All five a-e *SerpinA1* paralogs were deleted via CRISPR-Cas9 in *Serpina1* ^{Null} mouse (C57BL/6J-Serpina1em3Chmu/J knockout, strain #035015) from Jackson Laboratory.

Supplementary Figure S2

A

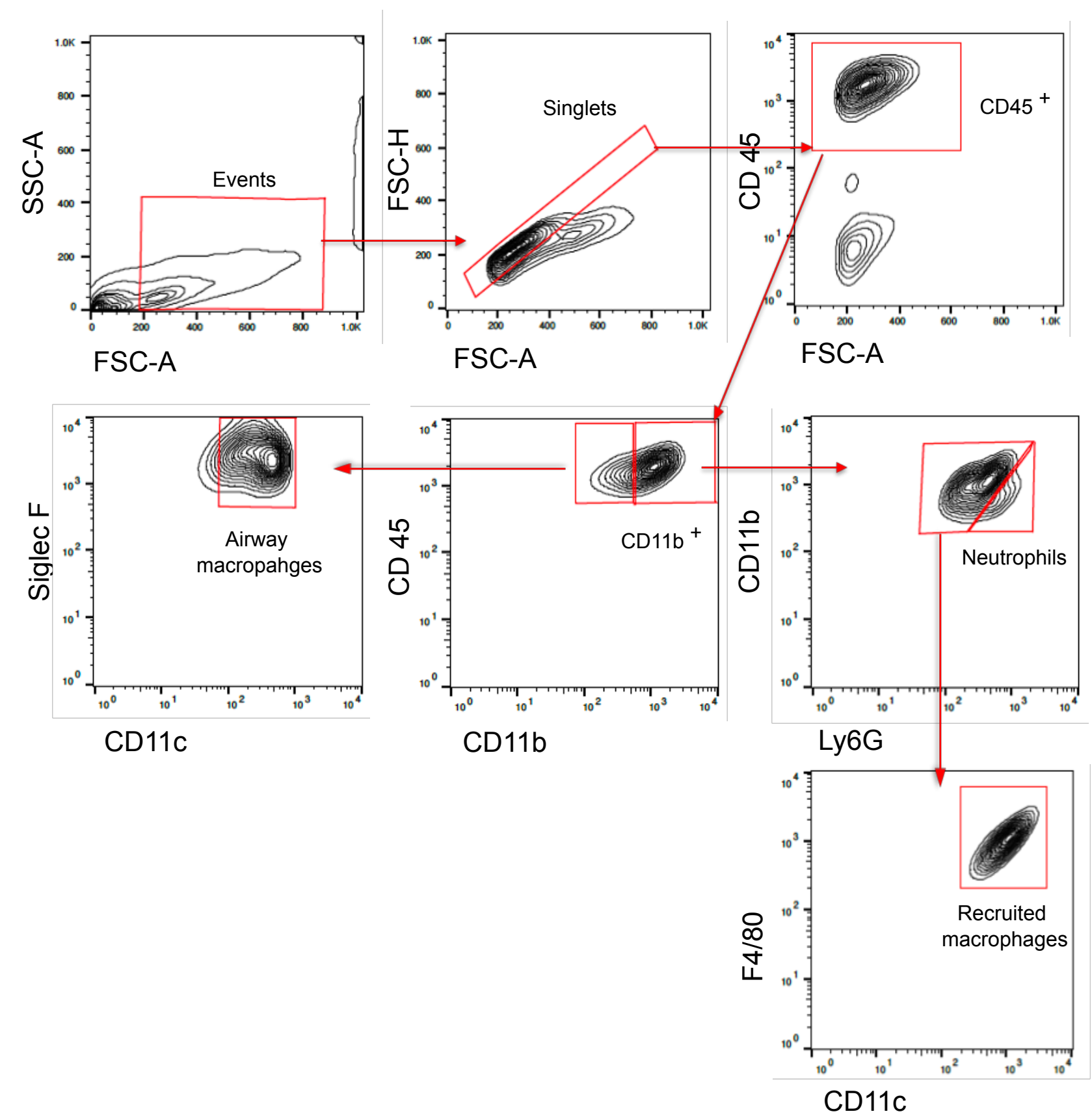

B

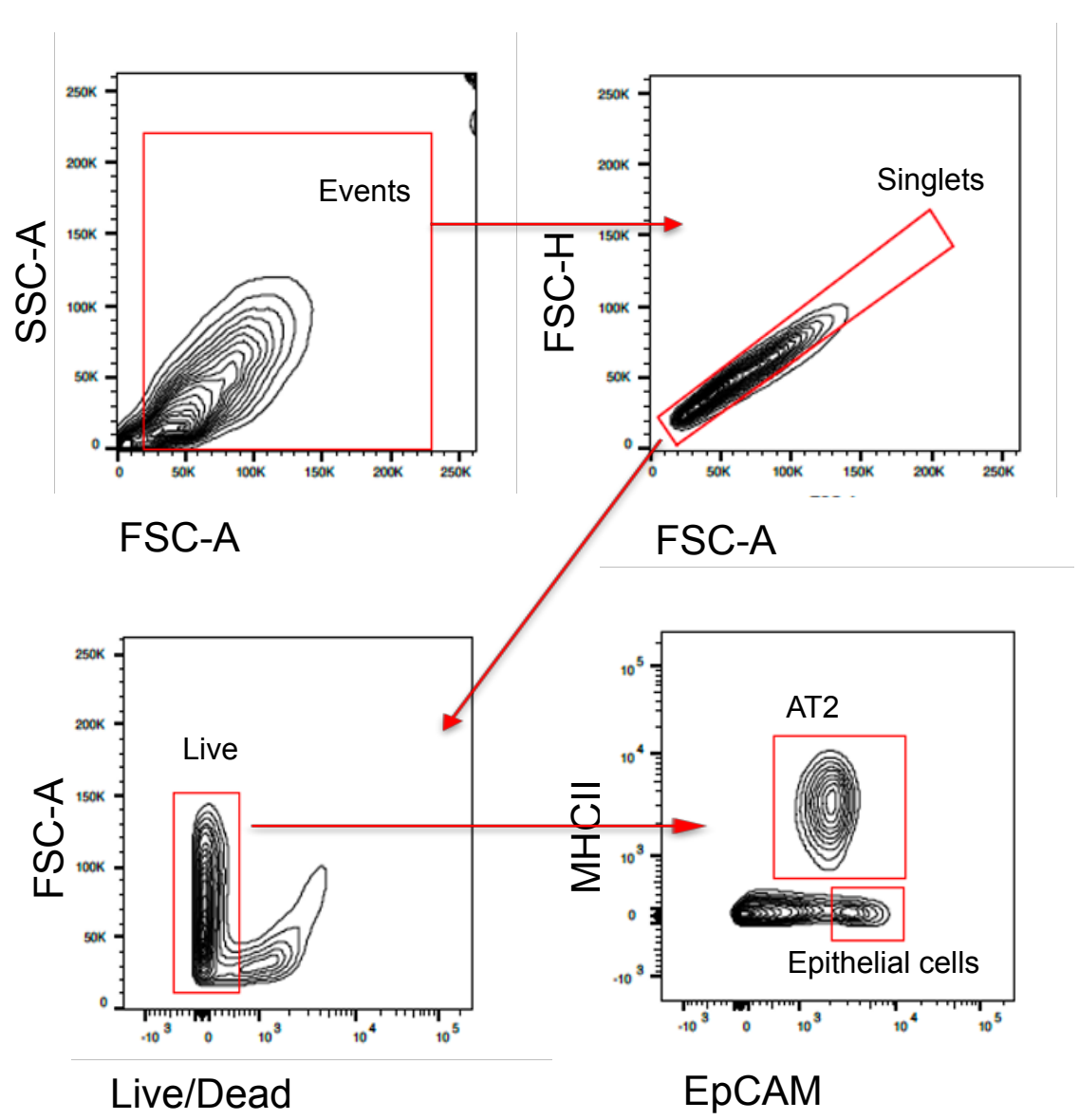

Supplementary figure S2. Gating strategy to identify inflammatory cells in the WT mouse BAL fluid and primary murine AT2 cells. **A**. Single cells of the BAL cell pellet, after the exclusion of debris and doublets were identified by CD45 staining. A sequential gating strategy was used to identify populations expressing specific surface markers: $CD11b^-$ $F4/80^+$ $CD11c^+$ airway macrophages, $CD11b^+$ $Ly6G^+$ neutrophils, $CD11b^+$ $F4/80^+$ $CD11c^+$ monocyte – derived recruited macrophages. **B**. Single cell suspension obtained by enzymatic digestion of mouse lungs was subjected to flow cytometry and sorting after the exclusion of debris and doublets. A sequential gating strategy was used to sort for live, $EpCAM^+MHCII^+$ (AT2) cells. FSC - forward scatter, SSC – side scatter.

Supplementary Figure S3

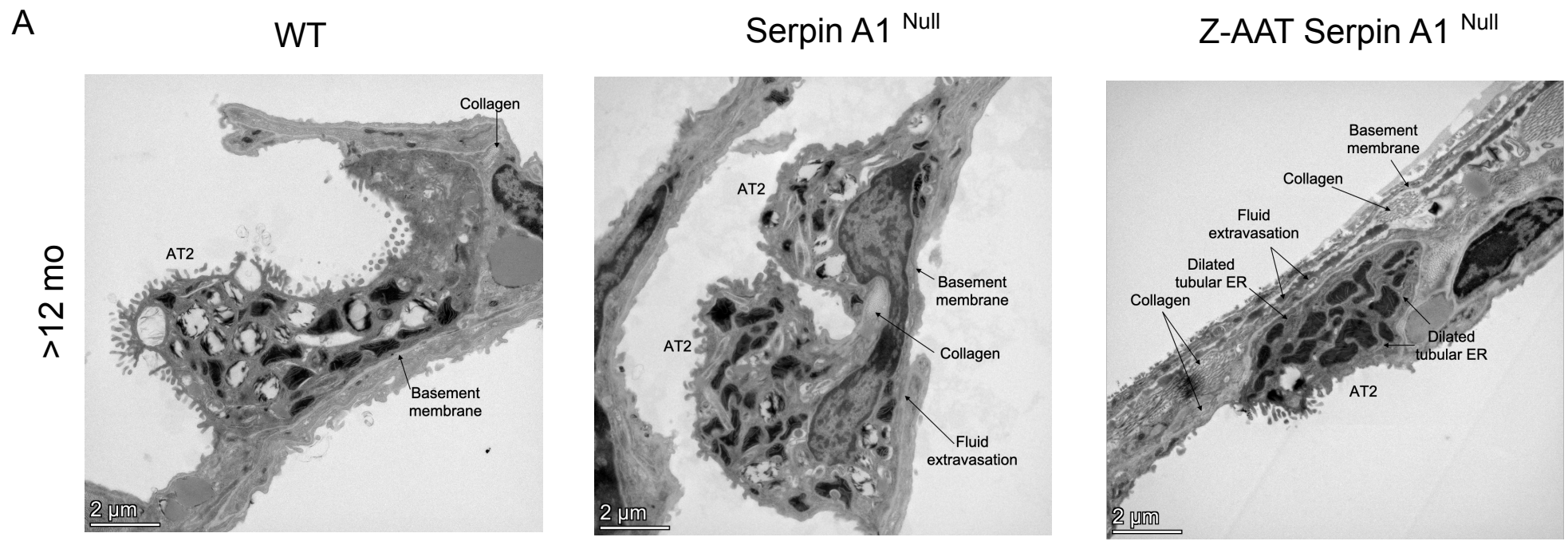

Supplementary figure S3 – TEM images of the alveolo-capillary unit. Representative TEM images of alveolo-capillary unit in Z-AAT *Serpina1* ^{Null}, *Serpina1* ^{Null} and WT mice; size bar 2 μm. Note thicker basement membrane, accumulation of fibrillar collagen, and fluid leak in the interstitial space in *Serpina1* ^{Null} and Z-AAT *Serpina1* ^{Null} mice, accompanied by fussed mitochondria and dilated tubular ER in Z-AAT *Serpina1* ^{Null} mice.

Supplementary Figure S4

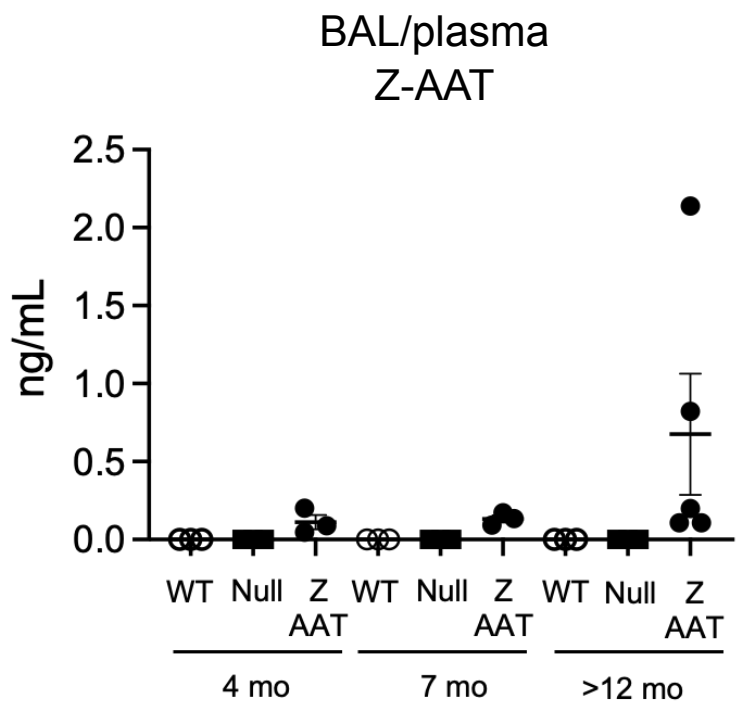

Supplementary figure S4. **BAL fluid to plasma Z-AAT polymer ratio**. Z-AAT levels measured by ELISA using 2C1 antibody in the BAL fluid and plasma of 4-, 7- and older than 12-month-old Z-AAT Serpina1 Null, Serpina1 Null and WT mice. Note the trend in higher BAL/plasma Z-AAT ratio in the older than 12-month-old Z-AAT mice, but not statistically different than 4-, 7-month-old Z-AAT mice. Data are presented as mean ± SD, 1-way ANOVA, followed by Tukey's multiple comparisons

Supplementary Figure S5

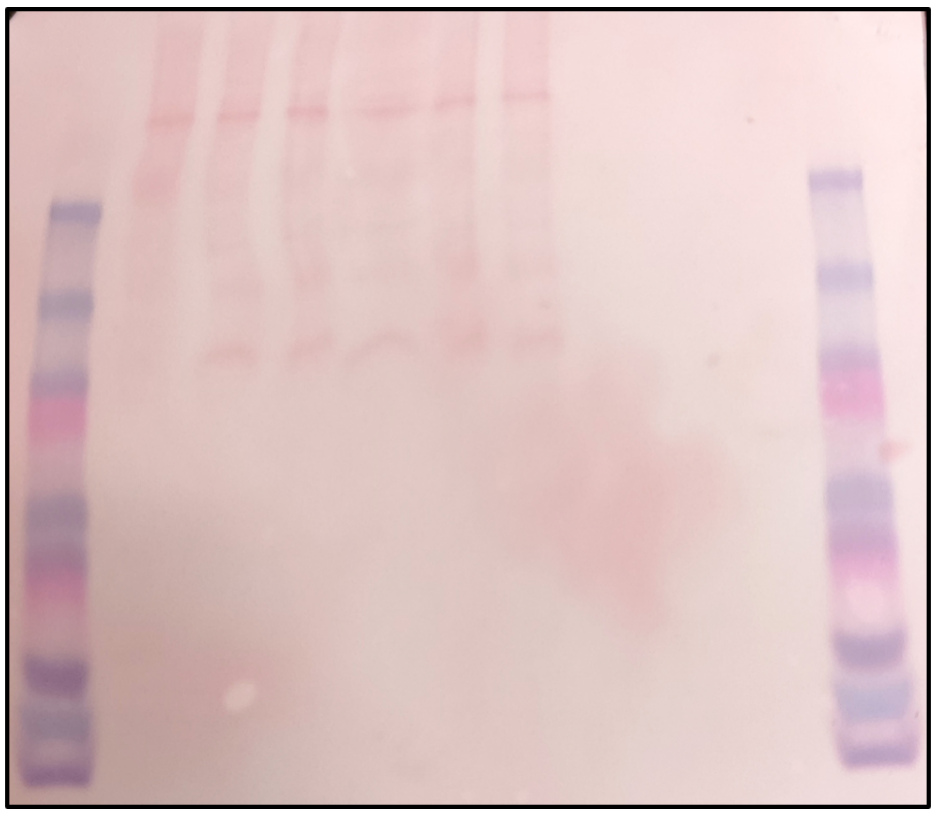

Supplementary figure S5. AT2 cells immunoblot Ponceau S staining. Immunoblot of whole cell lysates from primary murine AT2 cells of Z-AAT *Serpina1* ^{Null}, *Serpina1* ^{Null} and WT mice stained with Ponceau S to verify equal loading.

Supplementary Figure S6

A

Organoids Day 21

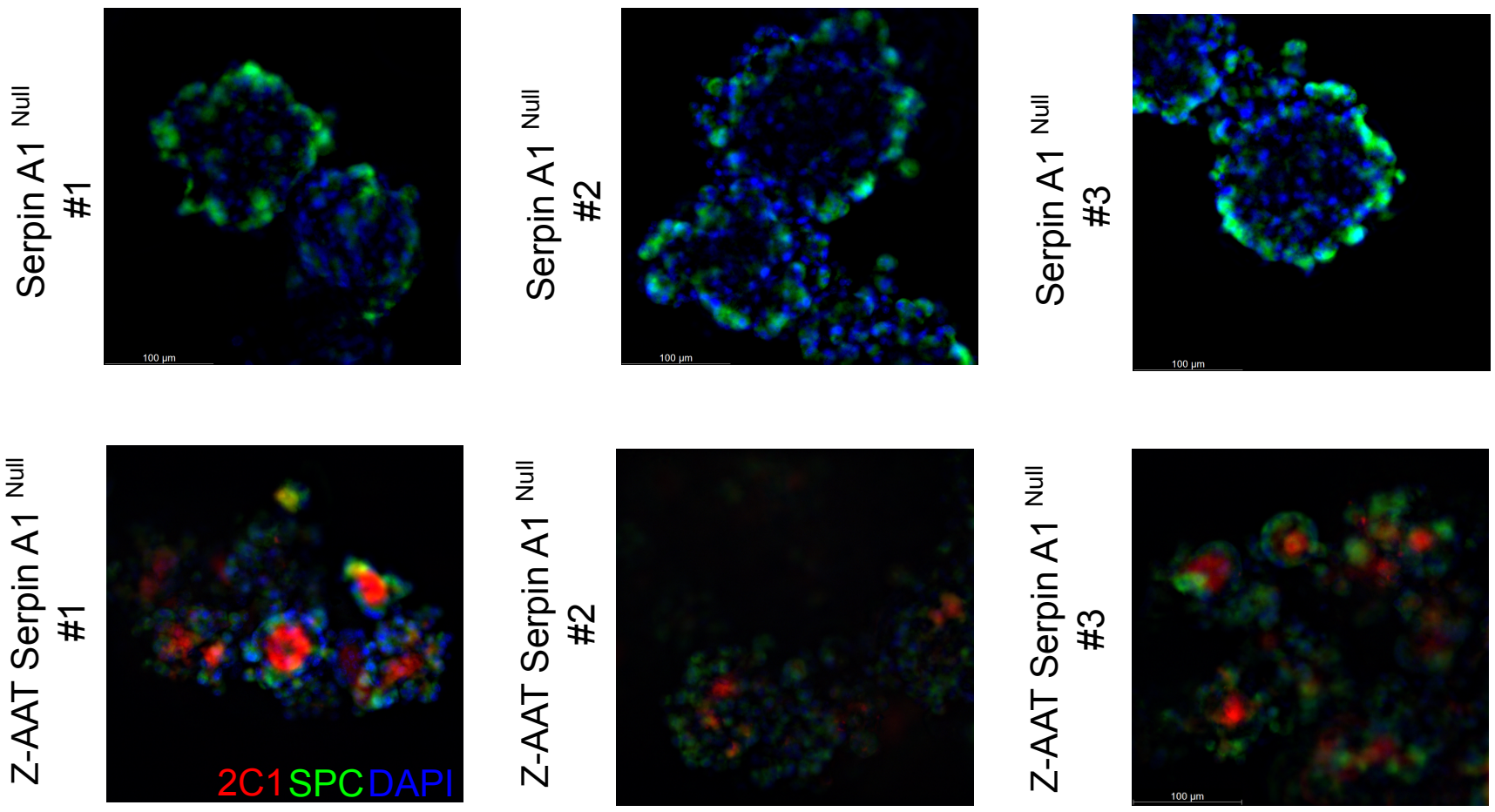

B

BAL cytoprep

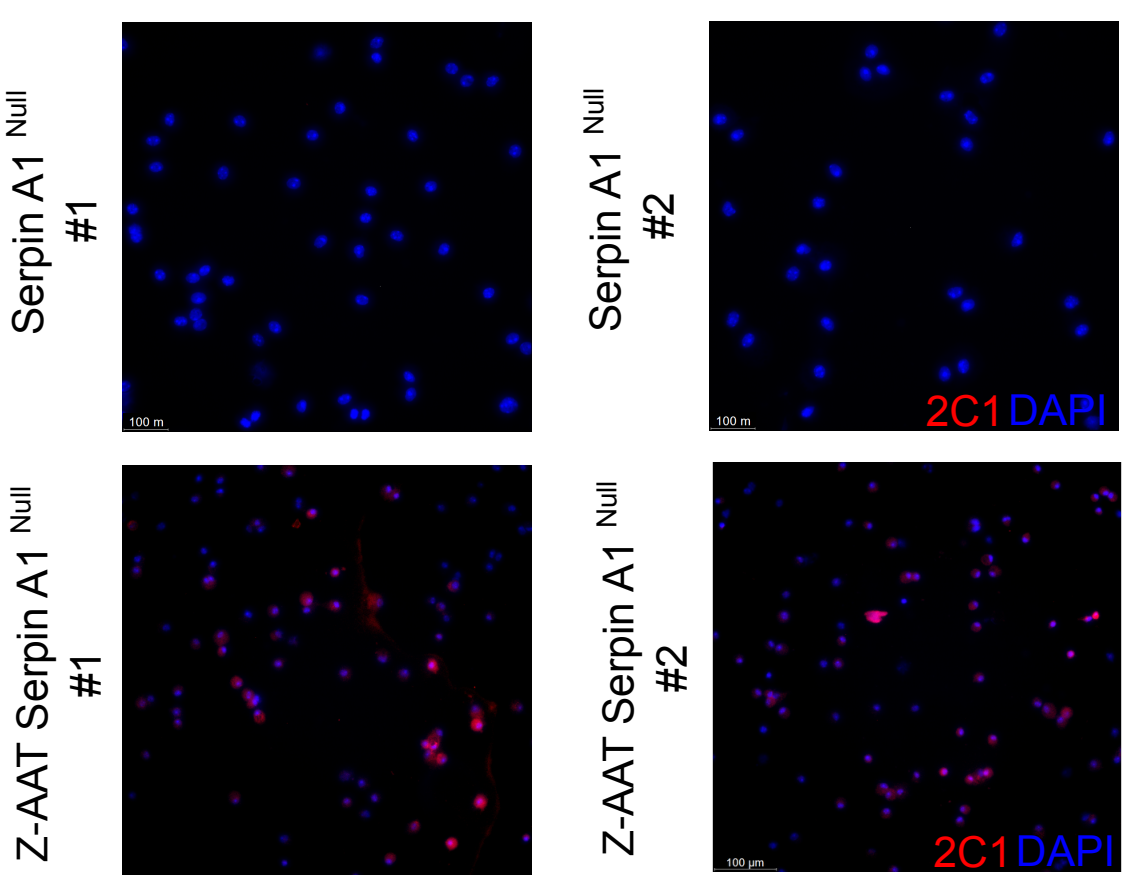

Supplementary figure S6. **Z-AAT polymers in AT2 organoids and murine macrophages**. **A**. Representative whole-mount immunofluorescent images of day 21 organoids stained with anti-SPC (green) and 2C1 (red) showing Z-AAT polymers secretion by differentiated AT2 from 3 different Z-AAT *Serpina1* ^{Null} vs. *Serpina1* ^{Null} mice, age 7-12-month-old; size bar 100µm **B**. Representative immunofluorescence images of Cytospin slides obtained from the BAL fluid of >12-month-old Z-AAT *Serpina1* ^{Null} vs. *Serpina1* ^{Null} mice after staining with 2C1 antibody (red, Hycult) and DAPI (blue), size bar 100µm.

Supplementary Figure S7

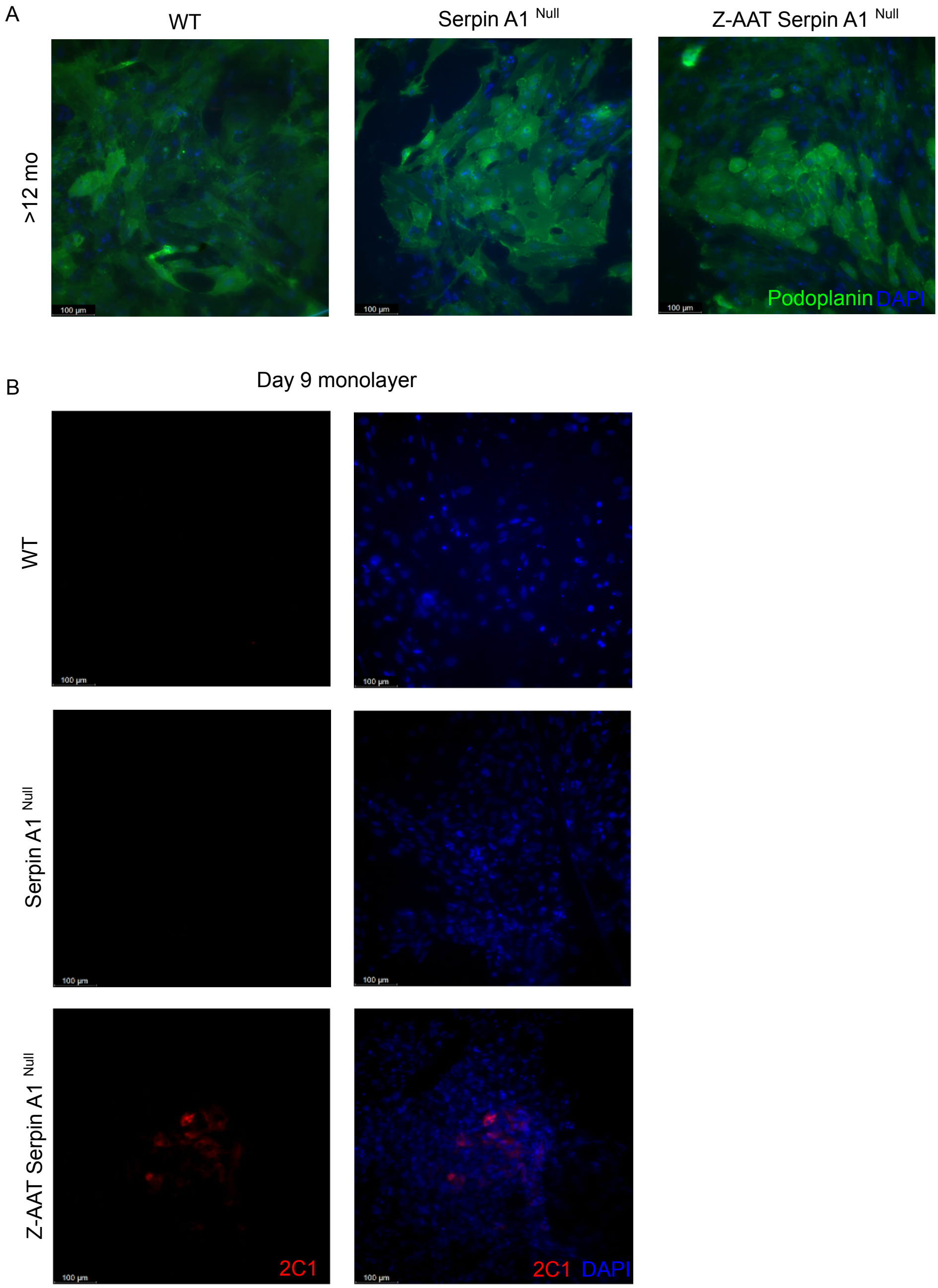

Supplementary figure S7. **Primary murine AT2 differentiation using a trans-well culture system secrete Z-AAT polymers. A.** Representative immunofluorescence images of Day 9 monolayers stained with an AT1 marker, podoplanin (green) and DAPI (blue) showing tight monolayer formation by differentiated AT2 cells of old WT mice grown in laminin-coated trans-wells. Note incomplete monolayer differentiated from AT2 cells of old Z-AAT *Serpina1* ^{Null} and *Serpina1* ^{Null} mice; scale 100 µm. **B**. Representative immunofluorescence images of Day 9 monolayers stained with 2C1 (red) and DAPI (blue) showing polymerized Z-AAT (red), size bar 100µm.

Supplementary Figure S8

A

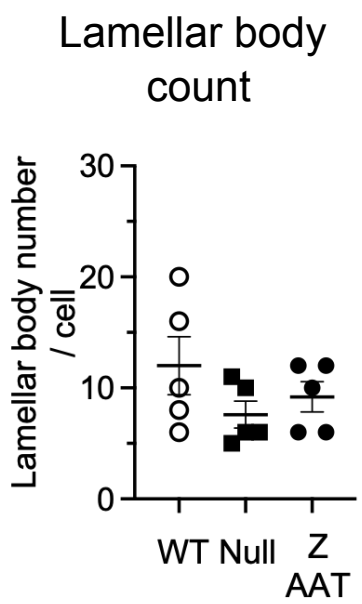

Supplementary figure S8 – TEM quantification of AT2 lamellar body abundance. Number of lamellar bodies per AT2 cell quantified by manual counting of 5 different TEM images from >12-month-old Z-AAT *Serpina1* ^{Null} vs. *Serpina1* ^{Null} and WT mice. Data are presented as mean ± SD, t-test.
