# Supplementary material for: Polymerized Z-α-1 antitrypsin leads to lung injury in a murine model of emphysema
Source: JCI Insight. 2026 Aug 10;11(15):e199029. doi: 10.1172/jci.insight.199029 (PMC13463610; doi:10.1172/jci.insight.199029)

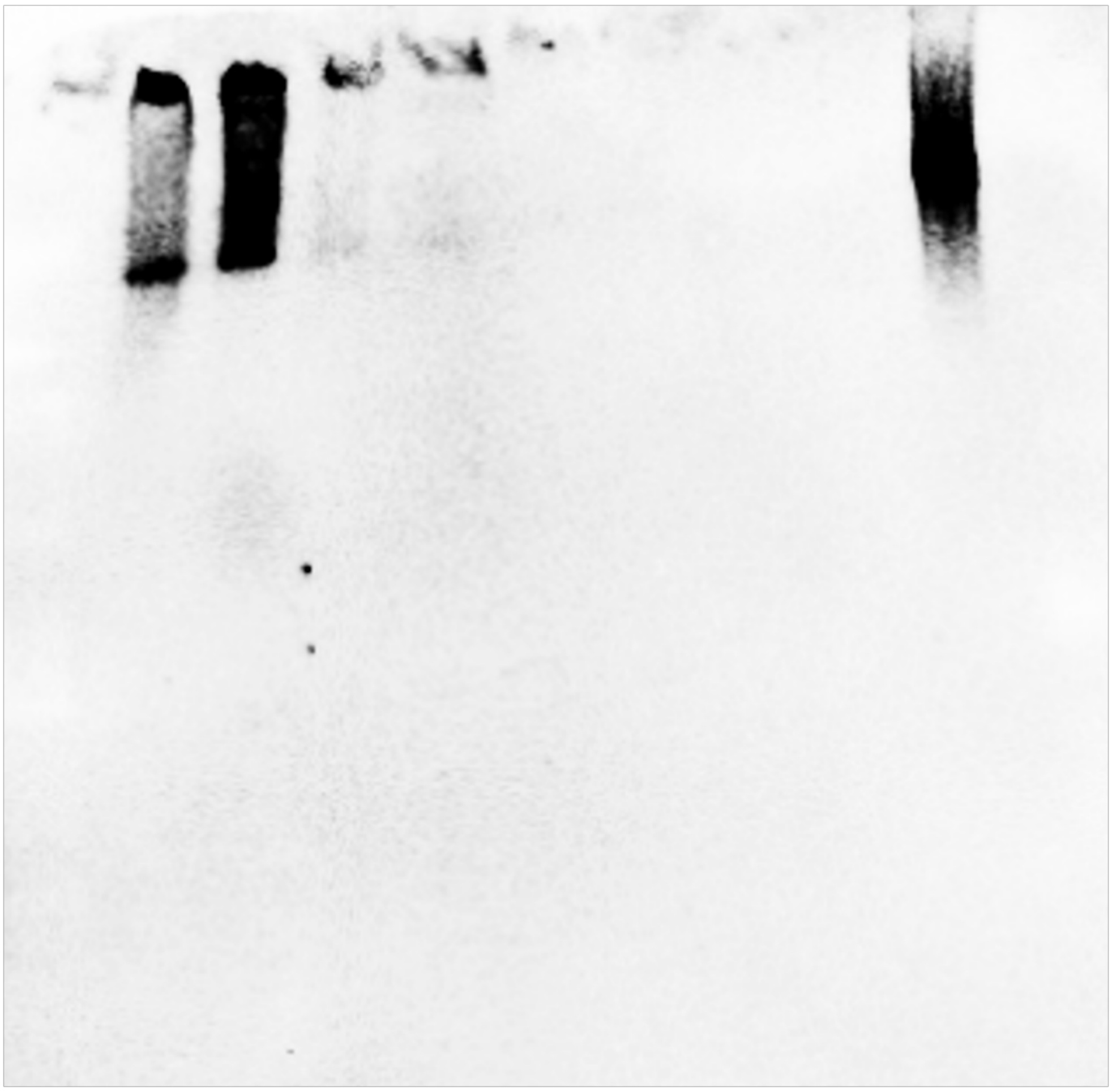

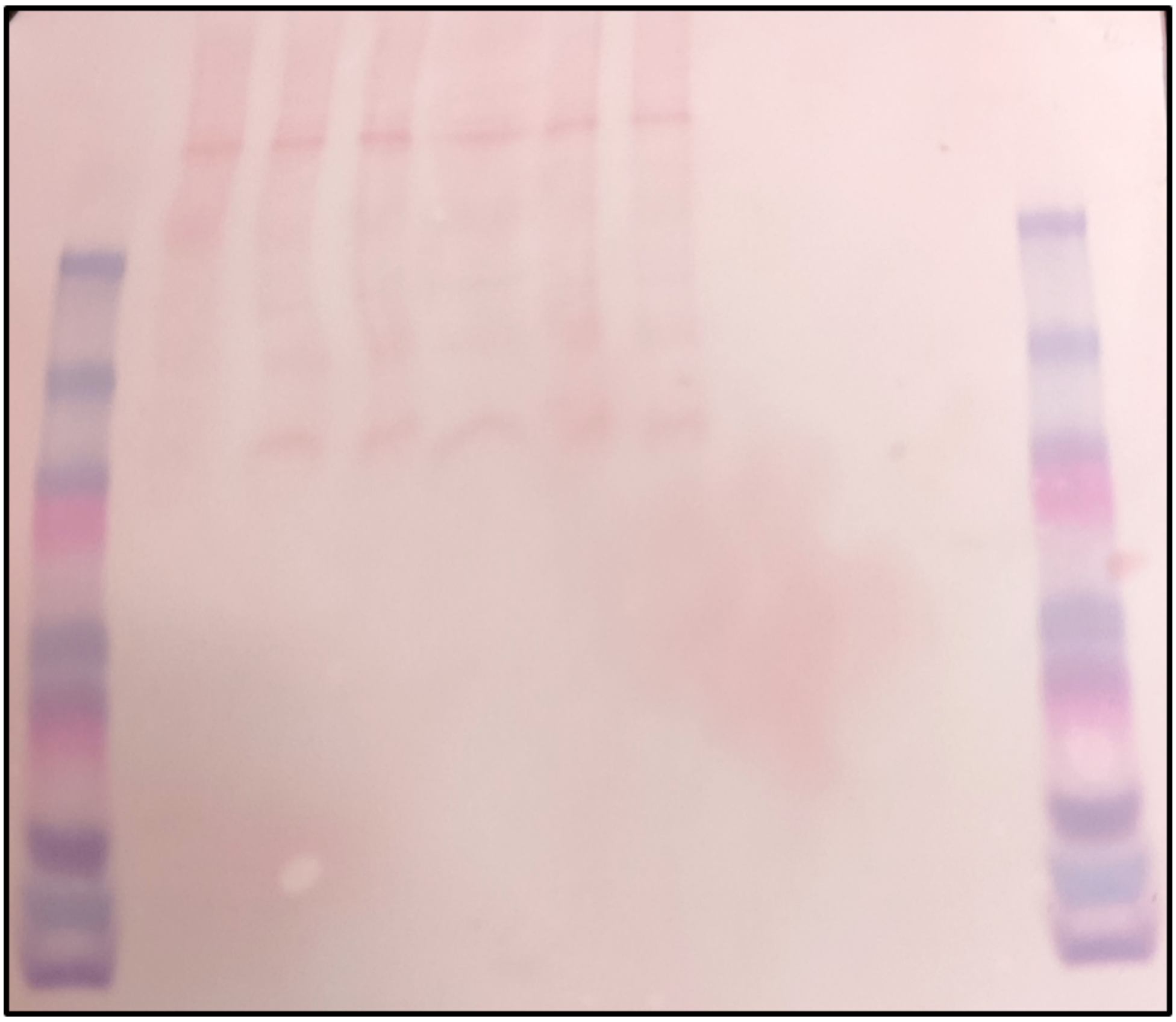

Supplement: Supplementary file 2 [file jciinsight-11-199029-s117.pdf]
